# C-Type Natriuretic Peptide Regulates the Expression and Secretion of Antibacterial Peptide S100A7 in Goat Mammary Gland Through PKG/JNK/c-Jun Signaling Pathway

**DOI:** 10.3389/fvets.2022.822165

**Published:** 2022-04-12

**Authors:** Mingzhen Fan, Yuyang Miao, Yutong Yan, Kunyuan Zhu, Xiaoe Zhao, Menghao Pan, Baohua Ma, Qiang Wei

**Affiliations:** ^1^Key Laboratory of Animal Biotechnology of the Ministry of Agriculture, Northwest A&F University, Xianyang, China; ^2^College of Veterinary Medicine, Northwest A&F University, Xianyang, China

**Keywords:** C-type natriuretic peptide, natriuretic peptide receptor B, antibacterial peptide, S100A7, mammary gland, mastitis, goat

## Abstract

During infection, the infected tissue secretes a variety of endogenous peptides to resist further invasion of pathogens. Among these endogenous peptides, the natriuretic peptides and the antimicrobial peptides attracted the most attention. C-type natriuretic peptide (CNP) and its receptor natriuretic peptide receptor B (NPR-B) were members of the natriuretic peptide system. The antimicrobial peptide S100A7 plays an important role to resist infection of bacteria in mastitis. It is reported that the expression of S100A7 is regulated by an activator protein-1 (AP-1)-responsive promoter. As a subunit of AP-1, c-Jun is a downstream target of CNP/NPR-B signaling pathway. Therefore, it is a hypothesis that the CNP/NPR-B signaling pathway induces the expression and secretion of S100A7 in mammary glands to take part in local mammary gland innate immunity. To verify this hypothesis, goat mammary gland and isolated mammary epithelial cells (MECs) were used to explore the expression of CNP/NPR-B and their physiological roles in goat mammary gland. The results showed that goat mammary gland expressed NPR-B, but not CNP. The expression and secretion of S100A7 in goat MECs were obviously induced by CNP/NPR-B signaling pathway. After treatment with CNP, the cyclic guanosine monophosphate (cGMP) level in goat MECs was significantly upregulated. Along with the upregulation of cGMP level, the phosphorylation levels of c-Jun N-terminal kinase (JNK) and its target c-Jun were also increased gradually. KT5823 is a specific inhibitor for protein kinase G (PKG). KT5823 remarkably inhibited the phosphorylation of JNK and c-Jun induced by CNP. Correspondingly, KT5823 evidently inhibited the expression and secretion of S100A7 induced by CNP. On the other hand, the expression of NPR-B and S100A7 was upregulated in the mastitis goat mammary gland. But, there was no significant difference in expression of CNP between healthy and mastitis goat mammary gland tissues. The goat mastitis model was established *in vitro* using goat MECs treated by lipopolysaccharide (LPS). LPS treatment also could increase the expression of NPR-B and S100A7. In conclusion, goat mammary gland expressed NPR-B, indicating mammary gland was the target organ for natriuretic peptide system. Moreover, CNP, through NPR-B/JNK/c-Jun signaling pathway to regulate the expression and secretion of S100A7 in MECs, played an important role in mammary gland innate immunity.

## Introduction

Mastitis causes huge economic losses in the milk industry in the world. One of the traditional methods for the prevention and treatment of mastitis is antibiotics. However, the use of antibiotics reduces the quality of milk and sometimes causes resistant microbes. The endogenous peptides produced by cells involved in animal innate immunity become valuable substitutes for antibiotics in the prevention and treatment of mastitis. Among these endogenous peptides, the natriuretic peptides (NPs) and the antimicrobial peptide (AMP) attracted the most attention (1–3).

The NP families, mainly including A-type natriuretic peptide (ANP), B-type natriuretic peptide (BNP), and C-type natriuretic peptide (CNP), are well known produced in the cardiovascular and central nervous systems (4). They play important roles in cardioprotective function, regulation of blood pressure, fluid balance, and follicles development (5, 6). CNP was first isolated from porcine brain (7). Its function was dependent on the activation of natriuretic peptide receptor B (NPR-B) [also termed guanylyl cyclase-B (GC-B)]. Once CNP binds to NPR-B, NPR-B induces the production of cyclic guanosine monophosphate (cGMP), thereby activating protein kinase G (PKG) and downstream targets (8). It is reported that CNP could suppress inflammation reactions. Activation of CNP/NPR-B signaling pathway downregulated the expression of proinflammatory factors, including interleukin-1β (IL-1β), interleukin-6 (IL-6), and tumor necrosis factor-α (TNF-α), and inhibited macrophage infiltration (9–13). Besides the anti-inflammation effect, CNP may directly affect bacteria lipopolysaccharide (LPS) biosynthesis (14) and biofilms formation (15–17).

S100A7 (Psoriasin), which is first identified in the epithelial cells of human psoriasis skin (18, 19), is one of the AMP with a strong antimicrobial activity, especially against *Escherichia coli* in humans (20) and bovine (21). In goat, S100A7 was expressed in the stratified squamous epithelium of the teat, gland cistern, and epithelial cells of the alveolus (22). Based on the antibacterial and immunomodulatory activities, the expression of S100A7 in the alveolus is considered to be a critical component of the animal innate immune system and plays an important role in the host mammary gland defense system (23). Many inflammatory cytokines such as IL-6, IL-17, IL-22, and TNF-α could upregulate the expression of S100A7 (24, 25). In human, the expression of S100A7 is regulated by an activator protein-1 (AP-1)-responsive promoter (26). As a subunit of AP-1, c-Jun was reported to be a target of natriuretic peptide signaling (27–29).

During infection, the infected tissue secretes a variety of endogenous peptides to form an innate immune microenvironment to resist further invasion of pathogens (17, 23, 30). Unlike ANP and BNP, CNP is usually regarded as an autocrine and paracrine factor to regulate local tissue function (8). Therefore, in this study, we verified a hypothesis that CNP/NPR-B signaling pathway induces the expression and secretion of S100A7 in mammary gland to take part in local mammary gland innate immunity. The results showed that goat mammary gland alveolus and mammary epithelial cells (MECs) expressed NPR-B. CNP/NPR-B regulated the expression and secretion of S100A7 *via* PKG/c-Jun N-terminal kinase (JNK)/c-Jun signaling pathway.

## Materials and Methods

### Animal

This study was approved by the Institutional Animal Ethical and Welfare Committee, Northwest A&F University, Shaanxi, China (Approval No. 2020039, Date 03.09.2020). Mammary gland tissue samples of healthy and mastitis goats were collected from lactating Guanzhong dairy goats in Shaanxi, China. The healthy goats (*n* = 6): no clinical signs and bacteriological tests negative and the mastitis goats (*n* = 6): evident clinical signs (udders were swollen and hard, there are floccules, clots or yellow color in the milk, and even stop lactation) and bacteriological tests positive. Both of the samples of healthy and mastitis goats were used to make paraffin sections and quantitative PCR. Only mammary gland tissues of healthy goats were used to isolate MECs.

### Goat Mammary Epithelial Cells Isolation and Culture

The mammary gland tissues were surgically isolated from lactating Guanzhong dairy goats. The mammary tissues were trimmed of visible fat and connective tissues and washed with phosphate-buffered saline (PBS) several times until the solution became pellucid and devoid of milk. The mammary tissues were minced into about 1 mm^3^ cubes and were then implanted into the 35-mm petri dish and were incubated at 37°C with saturated humidity and 5% CO_2_. After 6 h, 2 ml culture medium was added to the culture dish. The culture medium consisted of Dulbecco's Modified Eagle Medium/Nutrient Mixture F-12 (DMEM/F-12) (Invitrogen Corporation, Waltham, Massachusetts, USA) supplemented with 10% fetal bovine serum (Invitrogen Corporation, Waltham, Massachusetts, USA) and 100 IU/ml penicillin, 100 μg/ml streptomycin (Invitrogen Corporation, Waltham, Massachusetts, USA). The medium was replaced with fresh medium every 48 h until the cells migrated out of the tissue. About 10 days later, the cells spread across the bottom of the dish were passaged by digestion with 0.25% trypsin/ethylenediaminetetraacetic acid (EDTA). The cells were reseeded in a new 35-mm petri dish at a density of 2 × 10^5^ cells/cm^2^ and cultured at 37°C under 5% CO_2_. In present experiments, cells passaged within 9 times were used.

### Immunohistochemistry

The mammary tissues were fixed and kept in 4% paraformaldehyde (Solarbio, Beijing, China) at 4°C. After dehydrated and embedded in paraffin, a series of 3-μm sections were prepared and one of every five sections was selected for H&E staining or immunohistochemistry. Paraffin sections were deparaffinized and hydrated in graded ethanol series before staining with the streptavidin-peroxidase method. Antigens were retrieved by boiling for 15 min in Citrate Antigen Retrieval Buffer solution (Solarbio, China). Endogenous peroxidase was blocked by incubation in 3% hydrogen peroxide. Then, followed the instructions of the universal SP staining kit, in which the primary antibody anti-CNP (1:100), anti-NPR-B (1:100), and anti-S100A7 (1:100) (Bioss, Beijing, China) is incubated overnight at 4°C and the second antibody is incubated at 37°C for 1 h. After incubation, sections were lightly counterstained with H&E and were dehydrated and coverslipped. The immunohistochemical results were analyzed quantitatively using Image J software version 1.8.0.

### Immunofluorescence

Cells were fixed for 30 min in 4% paraformaldehyde (Solarbio, Beijing, China), permeabilized for 15 min in 0.2% Triton X-100 (Sigma, St. Louis, Mosby, USA), incubated for 2 h in blocking buffer [3% bovine serum albumin (BSA) in PBS], and treated with primary antibody against cytokeratin 18 (1:100, Abcam, Cambridge, Massachusetts, USA), cytokeratin 14 (1:100, Abcam, Cambridge, Massachusetts, USA), and NPR-B (1:100, Bioss, Beijing, China) overnight at 4°C. The secondary antibody used was goat antirabbit IgG-Alexa Fluor^®^ 488 (1:100, Abcam, Cambridge, Massachusetts, USA). The nuclei were stained with 4′,6-diamidino-2-phenylindole (DAPI) (Beyotime, Shanghai, China) for 5 min at room temperature. Images were captured using a fluorescence microscope (Olympus, Tokyo, Japan).

### Ribonucleic Acid Extraction and Reverse Transcription-PCR

Total RNA was extracted using RNAiso Plus (Takara Bio Incorporation, Otsu, Japan) according to the manufacturer's instructions. Complementary DNA (cDNA) was synthesized using the PrimeScript™ RT Reagent Kit (Takara Corporation, Dalian). The primer sequences are shown in Supplementary Table S1. The amplified products were separated and analyzed by electrophoresis on a 1% agarose gel. The quantitative reverse transcription-PCR (RT-PCR) reactions were performed with the Fast SYBR Green Master Mix (Genstar, Beijing, China); data collection and data analysis were performed on the QuantStudio 6 Flex Machine (Invitrogen Corporation, Waltham, Massachusetts, USA) by using the GraphPad Prism version 6 software. The quantitative RT-PCR parameters were as follows: 95°C for 2 min, followed by 40 cycles each of 95°C for 15 s, 60°C for 30 s, and 72°C for 30 s. Melt curve analysis and electrophoresis of amplified products confirmed that only a single product of the expected size was generated. All the samples run in triplicate reactions. Relative quantities of the amplified products were calculated according to 2^−ΔΔCT^ method. Relative gene expression was obtained by normalizing with glyceraldehyde-3-phosphate dehydrogenase (GAPDH) expression, calculating differences in messenger RNA (mRNA) expression as fold changes relative to expression in the control group or the healthy group.

### Western Blot Analysis

Cells were lysed with the RIPA buffer (Solarbio, Beijing, China) supplemented with 1 mM phenylmethylsulfonyl fluoride (Solarbio, Beijing, China) on ice for 30 min. Western blot was carried out with equal amounts of protein. The primary antibodies were as follows: anti-NPR-B (1:1,000, Bioss, Beijing, China), anti-S100A7 (1:1,000, Bioss, Beijing, China), anti-JNK (1:1,000, CST, Danvers, Massachusetts, USA), anti-phospho-JNK (1:1,000, CST, Danvers, Massachusetts, USA), anti-c-Jun (1:1,000, CST, Danvers, Massachusetts, USA), anti-phospho-c-Jun (1:1,000, CST, Danvers, Massachusetts, USA), and anti-GAPDH (1:1,000, CST, Danvers, Massachusetts, USA). The secondary antibodies used were goat antirabbit immunoglobulin G-horseradish peroxidase (IgG-HRP) (1:2,000, Abcam, Cambridge, Massachusetts, USA). The protein bands were detected with a chemiluminescence kit (Tanon, Shanghai, China).

### Enzyme-Linked Immunosorbent Assay

The cGMP and secretion of S100A7 from goat MECs were measured using ELISA. The cell lysate or cell culture medium was collected after treatment and stored at −20°C. The ELISA was performed using the Goat cGMP ELISA Kit and the Goat S100A7 ELISA Kit (Jianglaibio, Shanghai, China) according to manufacturer's protocol. The absorbance was measured at 450 nm using the microplate reader (Tecan, Tecan Group Ltd., Switzerland).

### Statistics

All the quantitative data are presented as mean ± SD with at least three biological replicates for each analysis. The average optical density and the Western blotting band gray intensity were measured by Image J software. SPSS software version 10.0 was used for data analysis. The paired samples *t*-test was applied to compare between the each experimental group and the control group, respectively. Results of *P* < 0.05 were considered as statistically significant ^*^: *p* < 0.05 (compared to control); ^**^: *p* < 0.01 (compared to control). The one-way ANOVA and Tukey's test were applied in all the groups multiple comparisons. Different letters indicate significant differences between groups (*P* < 0.05).

## Results

### Goat Mammary Gland Expressed Natriuretic Peptide Receptor B, but Did Not Express C-Type Natriuretic Peptide

**Figure 1:**
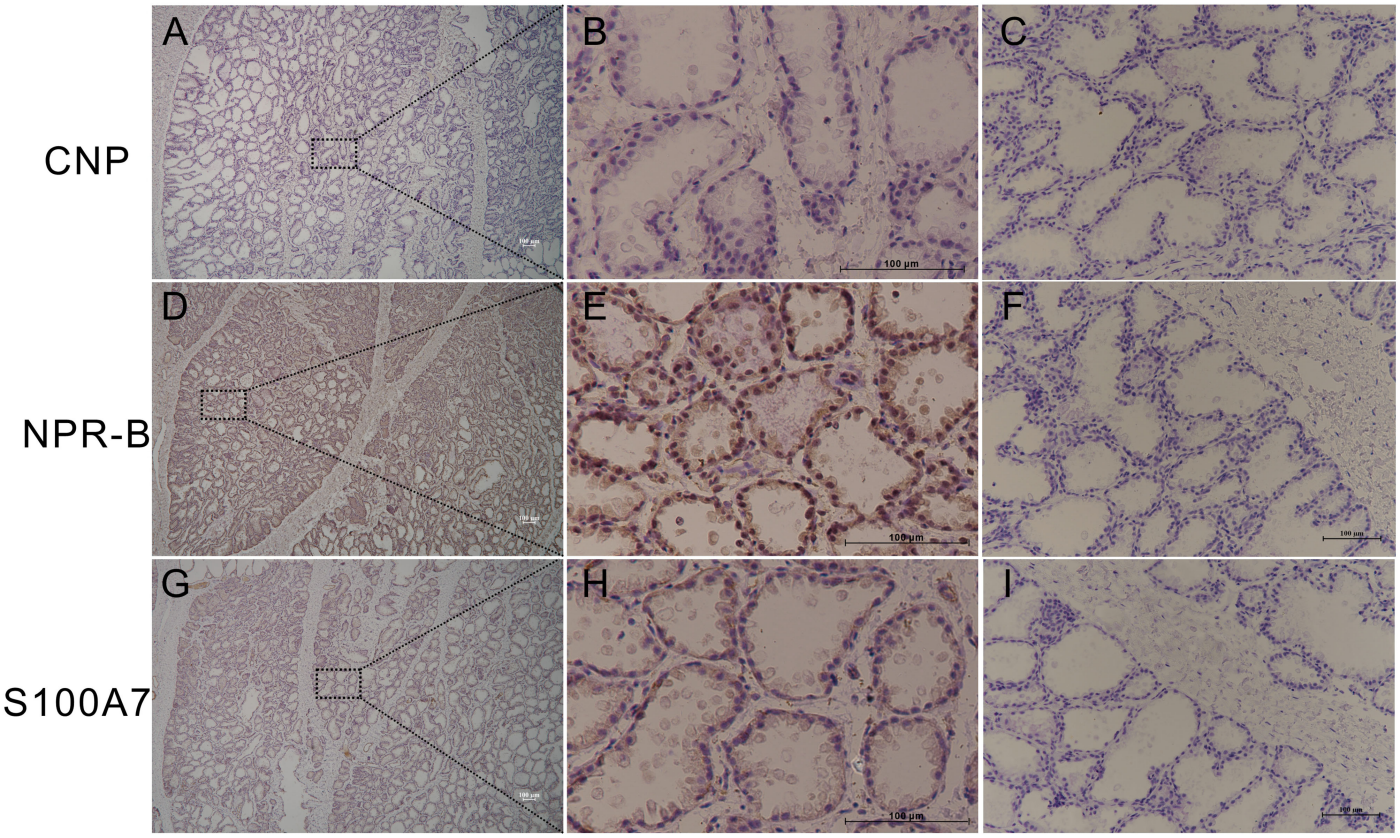
Representative images of immunohistochemistry in healthy goat mammary gland showing the expression of natriuretic peptide receptor B (NPR-B), but not C-type natriuretic peptide (CNP) in alveolus. **(A–C)** Immunohistochemical results of CNP. **(D–F)** Immunohistochemical results of NPR-B. **(G–L)** Immunohistochemical results of S100A7. **(C,F,L)** Negative control. Bar = 100 μm.

In order to study whether goat mammary gland expressed NPR-B and its ligand CNP, healthy goat mammary glands were used for immunohistochemical analysis. The results showed that the mammary lobules and alveolus were well developed in lactating goat mammary glands, NPR-B but not CNP positive immunoreactivity was present in the alveolus epithelial cells (Figures 1A–F). Only weak positive immunoreactivity could be observed in the alveolus epithelial cells (Figures 1G–L).

**Figure 2:**
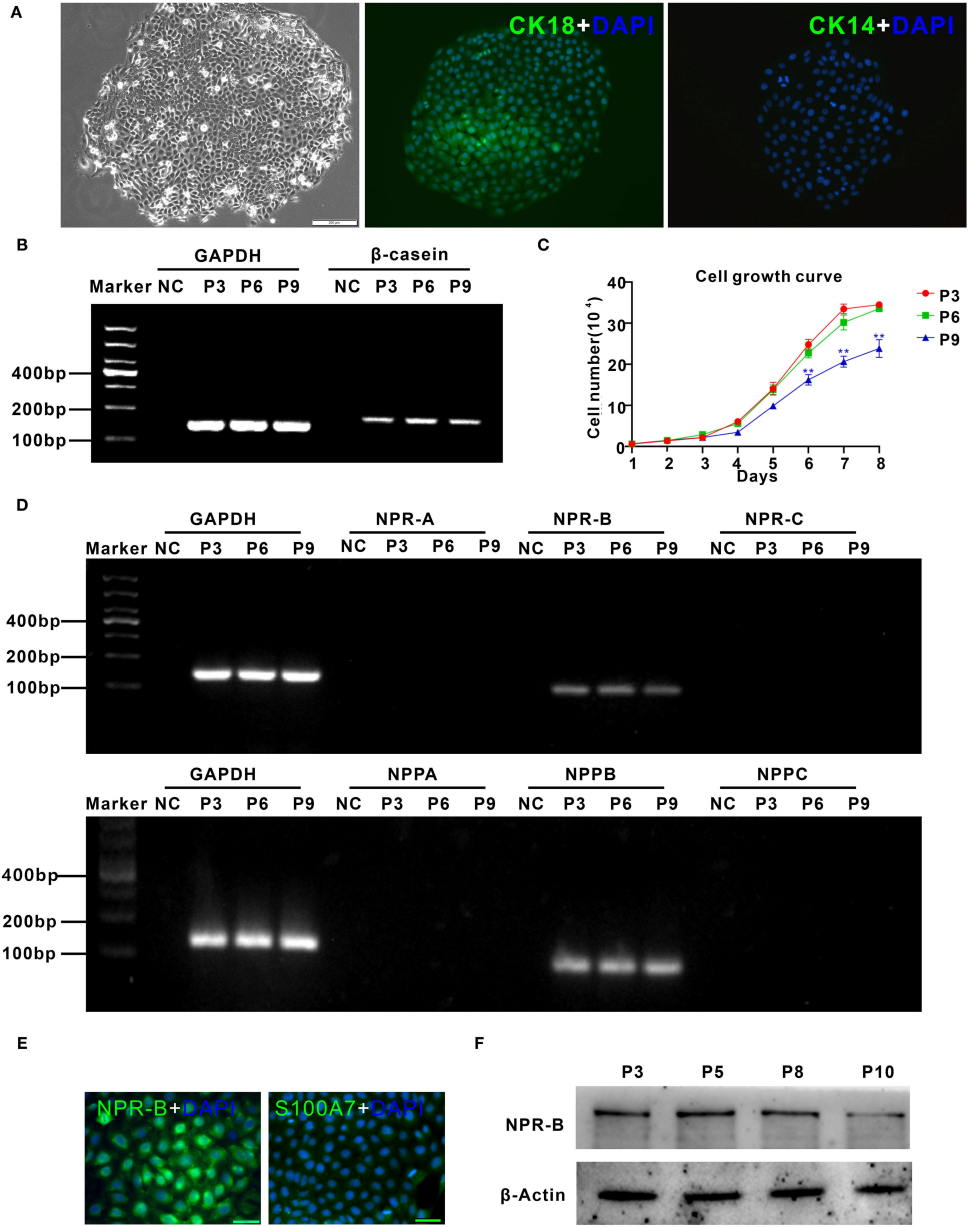
Characterization of goat mammary epithelial cells (MECs) and the expression of natriuretic peptides and natriuretic peptide receptors in goat MECs. **(A)** Characterization of goat MECs. Left, morphology of the isolated goat MECs (P5); middle, immunofluorescence result of cytokeratin 18 (CK18) in goat MECs; right, immunofluorescence result of cytokeratin 14 (CK14) in goat MECs. **(B)** Goat MECs at passages 3, 6, and 9 expressed β-casein detected by reverse transcription-PCR (RT-PCR). **(C)** Cell growth curve of goat MECs at passages 3, 6, and 9. **(D)** Goat MECs at passaged 3, 6, and 9 expressed NPPB messenger RNA (mRNA) and NPR-B mRNA, but not NPPA, NPPC, NPR-A, and NPR-C mRNA detected by RT-PCR. **(E)** Immunofluorescence result of NPR-B and S100A7 in goat MECs. **(F)** Goat MECs at passages 3, 6, and 9 expressed NPR-B detected by Western blotting. Full-length blots are given in Supplementary Figures S4, S5.

To further confirm the expression of NPR-B in mammary gland, goat MECs were isolated and cultured *in vitro*. Isolated goat MECs possessed typical epithelial cell morphology, including colony forming and cobblestone-like shape (Figure 2A). Immunofluorescence results showed that the isolated cells expressed luminal epithelial cells marker cytokeratin 18 (Figure 2A), but not myoepithelial cells marker cytokeratin 14 (Figure 2A). Furthermore, the messenger RNA (mRNA) of β-casein could be detected by RT-PCR (Figure 2B) in isolated cells. These results indicated that the isolated cells were goat mammary epithelial cells. Cell growth curve results showed that the isolated goat MECs proliferated vigorously within 9 passages (Figure 2C). RT-PCR results indicated that goat MECs expressed the mRNAs of NPR-B and BNP precursor (NPPB). However, the mRNAs of the other two receptors, namely, NPR-A and NPR-C and the other two natriuretic peptide precursors, namely, NPPA and NPPC, could not be detected (Figure 2D). The expression of NPR-B mRNA without NPPC mRNA was consistent with the immunohistochemical results mentioned above. Moreover, the immunofluorescence and Western blotting results further confirmed the expression of NPR-B in goat MECs (Figures 2E,F). However, the fluorescence intensity of S100A7 in goat MECs was very weak (Figure 2E).

### C-Type Natriuretic Peptide Induced the Expression and Secretion of S100A7 in Goat Mammary Epithelial Cells

Although goat MECs expressed NPR-B, the role of NPR-B in the physiological function of MECs is unclear. CNP, a high affinity ligand of NPR-B, was added into MECs culture medium at different concentrations. As the main functions of MECs, the effect of CNP on synthesis of β-casein and fatty acid was first analyzed. As shown in Supplementary Figure S1, CNP had no effect on mRNA expression of β-casein (Supplementary Figure S1A). The genes related to synthesis of fatty acids such as acetyl-CoA carboxylases alpha (ACACA) and sterol regulatory element-binding transcription factor 1 (SREBF1) were also not affected by CNP treatment (Supplementary Figure S1A). Meanwhile, Oil Red O stained results showed that CNP did not affect the triglyceride synthesis in goat MECs (Supplementary Figure S1B). On the other hand, cell proliferation and cell cycle analysis results indicated that CNP had no effect on cell proliferation of MECs (Supplementary Figure S2).

**Figure 3:**
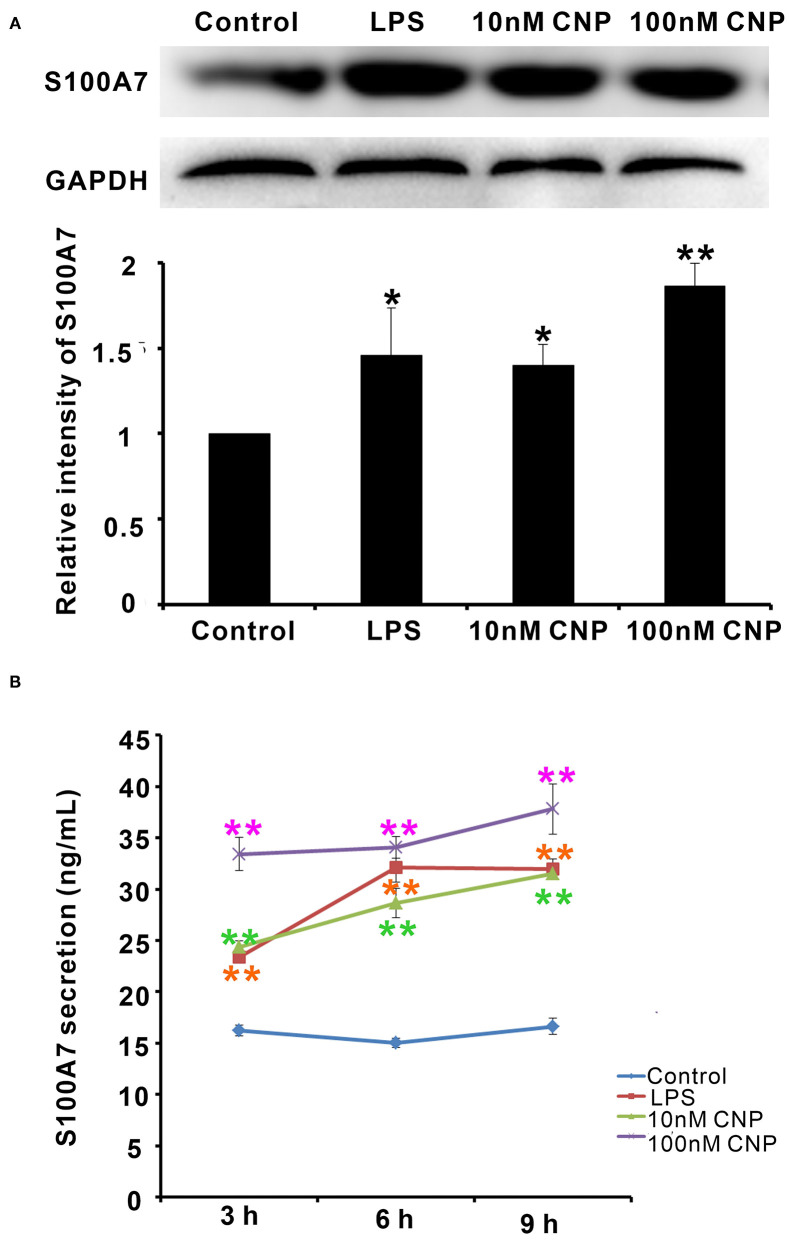
CNP induced the expression and secretion of S100A7 in goat MECs. **(A)** Western blotting results showed the protein expression level of S100A7 in goat MECs after treatment by lipopolysaccharide (LPS), 10 nM CNP, and 100 nM CNP, respectively, for 6 h. **(B)** ELISA results showed the S100A7 concentration in medium in which goat MECs were cultured and treated by LPS, 10 nM CNP, and 100 nM CNP, respectively, for 3, 6, and 9 h. *p < 0.05 (compared to control); **p < 0.01 (compared to control). Full-length blots are given in Supplementary Figures S6, S7.

Interestingly, the expression and secretion of antimicrobial peptide S100A7 in goat MECs were obviously induced by CNP treatment. Like positive control LPS, goat MECs treated by 10 nM CNP for 6 h, the protein expression level of S100A7 significantly increased compared with control (0 nM CNP) (*p* < 0.05) (Figure 3A). 100 nM CNP treatment for 6 h, the protein expression level of S100A7 remarkably upregulated compared with control (0 nM CNP) (*p* < 0.01) (Figure 3A). In addition to protein expression, the secretion of S100A7 from goat MECs into the medium was also tested by ELISA. The results showed that like positive control LPS, 10 nM and 100 nM CNP treatment only for 3 h, the secretion of S100A7 was evidently increased compared to control (0 nM CNP) (*p* < 0.01) and the increase in S100A7 concentration in medium was consistently maintained until treatment for 9 h in present experiments (Figure 3B). These results indicated that CNP could induce the expression and secretion of S100A7 in goat MECs.

### C-Type Natriuretic Peptide Activated Natriuretic Peptide Receptor B/c-Jun N-Terminal Kinase/c-Jun Signaling Pathway in Goat Mammary Epithelial Cells

**Figure 4:**
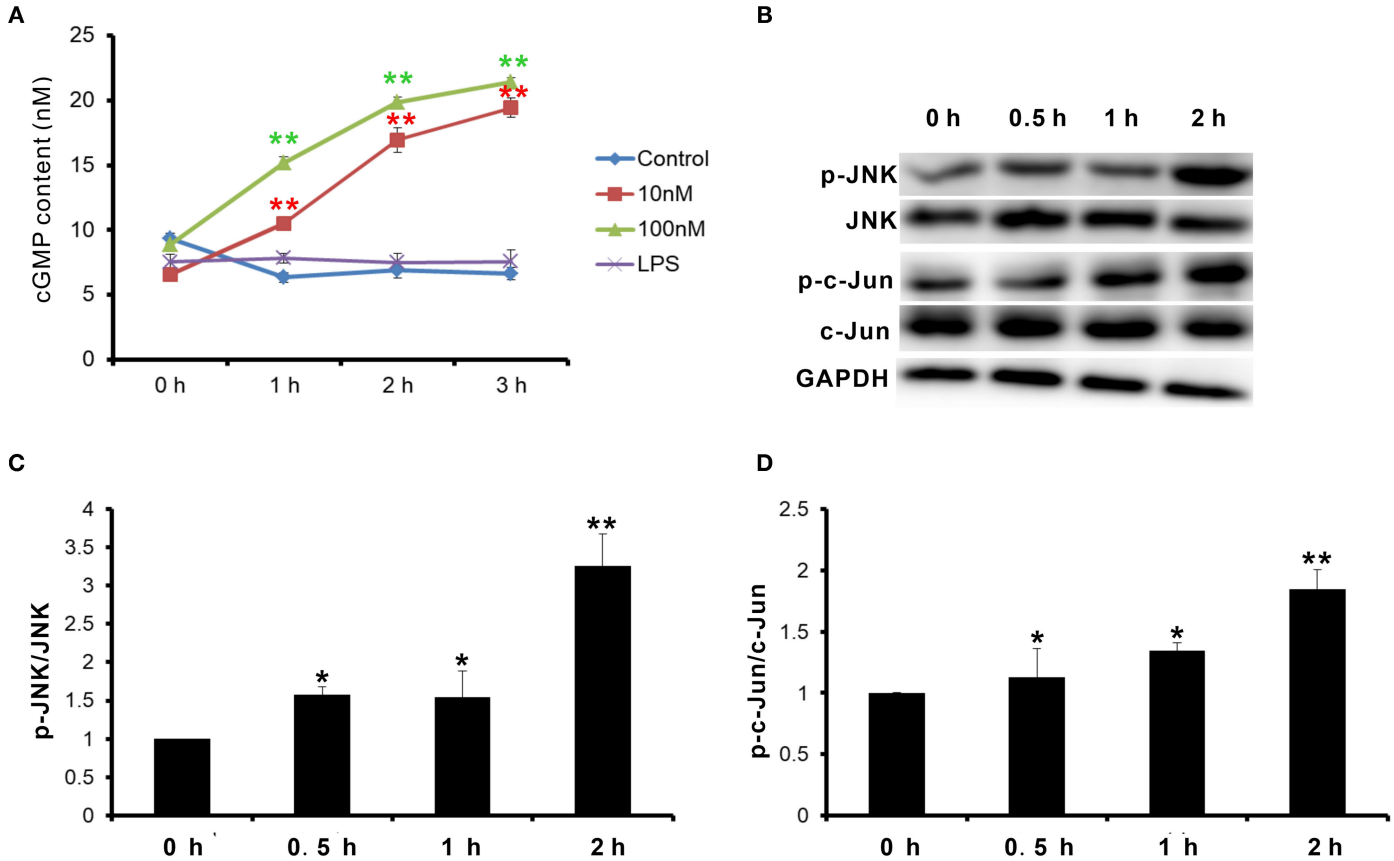
CNP activated NPR-B/c-Jun N-terminal kinase (JNK)/c-Jun signaling pathway in goat MECs. **(A)** ELISA results showed the cGMP content in goat MECs after treatment by 0 (control), 10, and 100 nM CNP or LPS for 0, 1, 2, and 3 h, respectively. **(B)** The phosphorylation levels of JNK and c-Jun in goat MECs after treatment by 0 (control), 10, and 100 nM CNP for 0, 0.5, 1, and 2 h, respectively, detected by Western blotting. **(C)** The quantitative analysis results of relative phosphorylation level of JNK in goat MECs after treatment by 0 (control), 10, and 100 nM CNP for 0, 0.5, 1, and 2 h, respectively. **(D)** The quantitative analysis results of relative phosphorylation level of c-Jun in goat MECs after treatment by 0 (control), 10, and 100 nM CNP for 0, 0.5, 1, and 2 h, respectively. *p < 0.05 (compared to control); **p < 0.01 (compared to control). Full-length blots are given in Supplementary Figures S8–S12.

Natriuretic peptide receptor B is a guanylate cyclase coupled receptor. Its physiological function depends on the guanylate cyclase activity, which cyclizes guanosine 5′-triphosphate (GTP) into cGMP. cGMP acts as the second messenger to activate the downstream signal pathway. Therefore, first, the change of cGMP content in goat MECs after CNP or LPS treatment was detected. After treatment with 10 nM and 100 nM CNP for 1–3 h, the cGMP levels in goat MECs were significantly upregulated compared to control (0 nM CNP) (*p* < 0.01) (Figure 4A). But, no significant difference was shown between LPS and control (0 nM CNP) (*p* > 0.05) (Figure 4A). Moreover, along with the upregulation of cGMP level, compared with control (0 nM CNP), the phosphorylation level of JNK also increased gradually after 100 nM CNP treatment (*p* < 0.05) (Figures 4B,C). As a target protein of JNK, after 100 nM CNP treatment, the phosphorylation level of c-Jun was upregulated significantly compared with control (0 nM CNP) (*p* < 0.05) (Figures 4B,D). These results indicated that CNP could activate NPR-B/JNK/c-Jun signaling pathway in goat MECs.

### Protein Kinase G Inhibitor KT5823 Inhibited the Expression and Secretion of S100A7 Induced by C-Type Natriuretic Peptide in Goat Mammary Epithelial Cells

**Figure 5:**
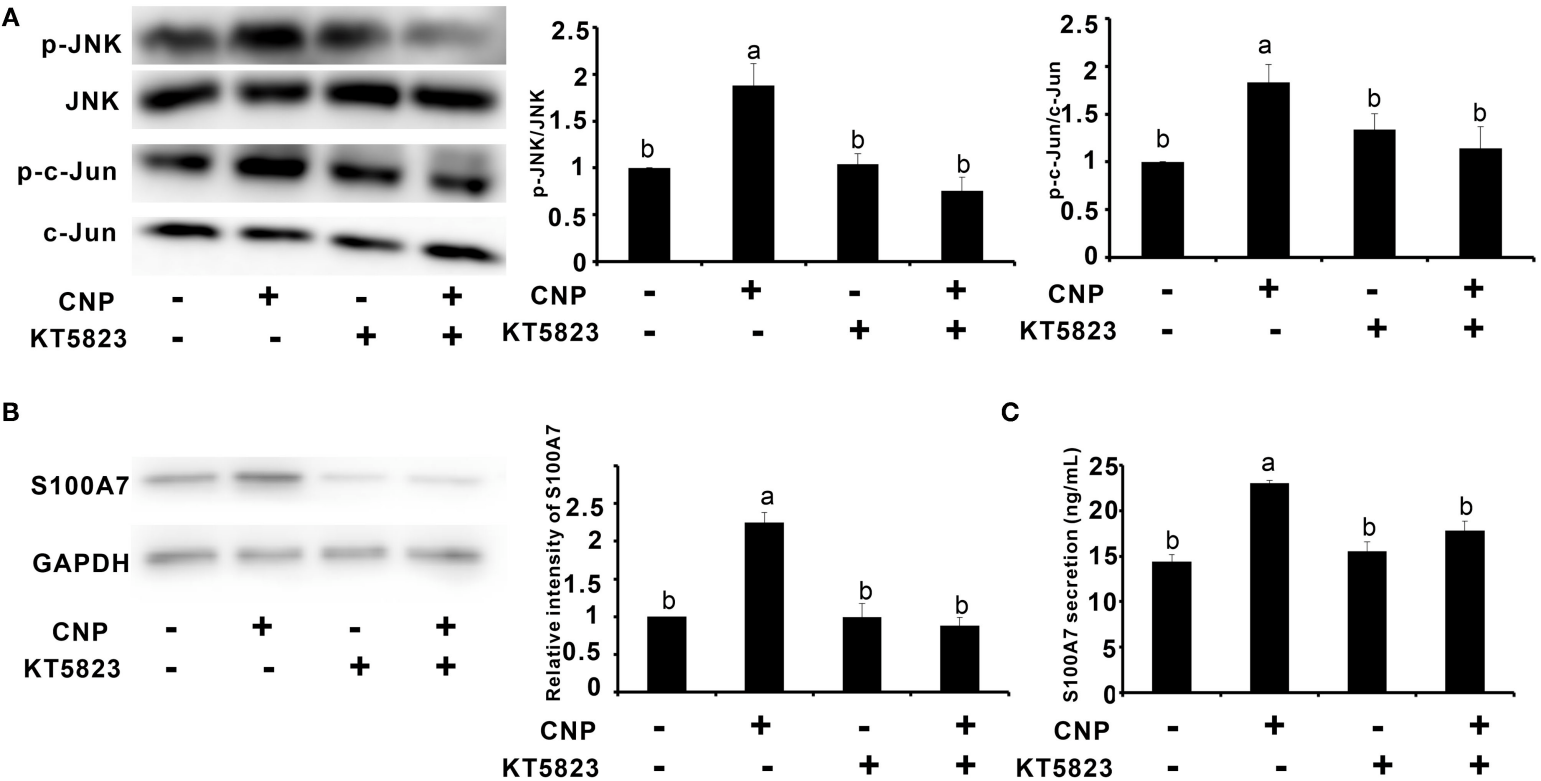
Protein kinase G (PKG) inhibitor KT5823 inhibited the expression and secretion of S100A7 induced by CNP in goat MECs. **(A)** The Western blotting and quantitative analysis results of relative phosphorylation level of JNK and c-Jun in goat MECs after treatment with 100 nM CNP alone or both of 100 nM CNP and 1 μM KT5823 simultaneously for 2 h. **(B)** The Western blotting and quantitative analysis results of S100A7 expression in goat MECs after treatment with 100 nM CNP alone or both of 100 nM CNP and 1 μM KT5823 simultaneously for 6 h. **(C)** ELISA results showed the S100A7 concentration in medium in which goat MECs were cultured and treated by 100 nM CNP alone or both of 100 nM CNP and 1 μM KT5823 simultaneously for 6 h. Different letters indicate significant differences between the groups, *P* < 0.05. Full-length blots are shown in Supplementary Figures S13–S18.

Since CNP activated NPR-B/JNK/c-Jun signaling pathway in goat MECs, it is necessary to test whether the expression and secretion of S100A7 were dependent on this signaling pathway. KT5823 is a selective inhibitor for PKG (cGMP-dependent protein kinase, PKG). As shown in Figure 5, addition of CNP alone could significantly increase the phosphorylation levels of JNK and c-Jun compared to control (*p* < 0.05) (Figure 5A). However, KT5823 remarkably inhibited the phosphorylation of JNK and c-Jun induced by CNP (Figure 5A). Correspondingly, the expression level and secretion content of S100A7 were significantly increased by treatment with 100 nM CNP alone for 6 h compared to control (*p* < 0.05) (Figures 5B,C). If adding both the CNP and KT5823 simultaneously, KT5823 evidently inhibited the expression of S100A7 induced by CNP (*p* < 0.05) and S100A7 secreted from goat MECs into medium was also downregulated compared with treatment with CNP alone (*p* < 0.05) (Figures 5B,C). The inhibitory effect of KT5832 on inductive function of CNP suggested that CNP induced the expression and secretion of S100A7 in goat MECs depending on NPR-B/PKG/JNK/c-Jun signaling pathway.

### Expression of Natriuretic Peptide Receptor B and S100A7 Was Upregulated in Mastitis Goat Mammary Gland

**Figure 6:**
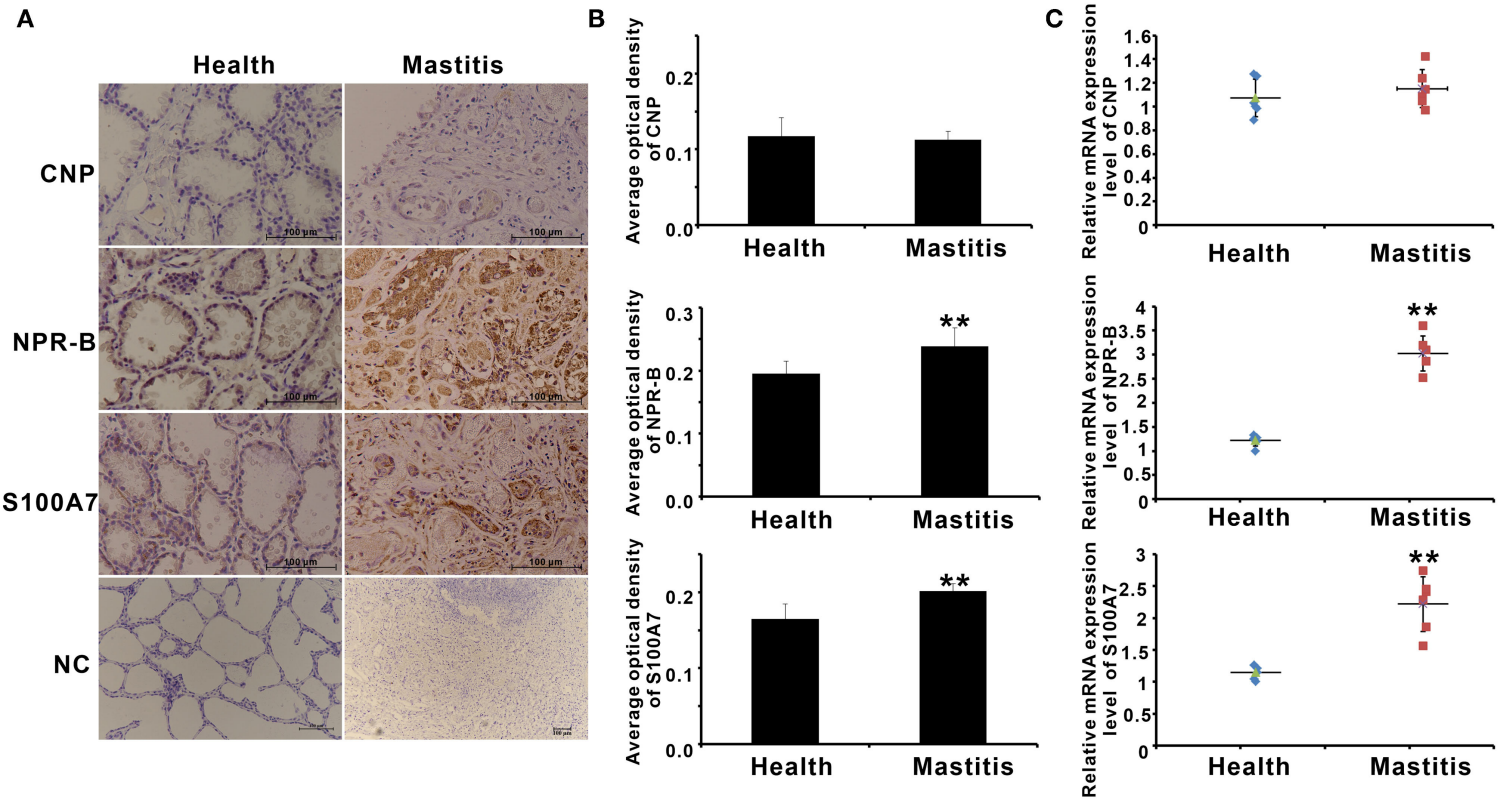
The expression of CNP, NPR-B, and S100A7 in healthy and mastitis goat mammary glands. **(A)** Representative images of immunohistochemistry showing the expression of CNP, NPR-B, and S100A7 in healthy and mastitis goat mammary glands. **(B)** Quantitative analysis of average optical density (AOD) of CNP, NPR-B, and S100A7 in healthy and mastitis goat mammary glands. **(C)**, Quantitative PCR results showing the mRNAs expression level of CNP, NPR-B, and S100A7 in healthy and mastitis goat mammary glands. ** *p* < 0.01 (compared to the healthy group).

S100A7 is an antimicrobial peptide with antimicrobial activities for a broad spectrum of bacteria. Therefore, it is a hypothesis that CNP/NPR-B, which induced goat MECs to express and secrete S100A7, may be involved in the occurrence or prevention of mastitis in goat. In order to verify this hypothesis, the expression of CNP, NPR-B, and S100A7 in healthy (*n* = 6) and mastitis (*n* = 6) goats was detected by immunohistochemistry and quantitative PCR. In healthy goat mammary gland, pathological examination results showed that the lobule and alveolus were well developed and completed (Supplementary Figure S3). Except for CNP, both the NPR-B and S100A7 were expressed in the alveolus epithelial cells in healthy goat mammary gland (Figure 6A). But, in mastitis goat mammary gland, pathological examination results showed that the alveolus collapsed and the infiltration of blood cells could be seen in the collapsed alveolus (Supplementary Figure S3). The immunohistochemical results showed that the densely NPR-B and S100A7 immunoreactivity could be observed in collapsed alveolus in mastitis mammary gland (Figure 6A). Like in healthy goat, CNP could not be detected in the alveolus epithelial cells in mastitis goat mammary gland (Figure 6A). The average optical density (AOD) of NPR-B and S100A7 in mastitis goat mammary gland was obviously higher than that in healthy goat mammary gland (*p* < 0.01) (Figure 6B). But, there was no significant difference in AOD of CNP between healthy and mastitis goat mammary glands (*p* > 0.05) (Figure 6B). Similarly, the mRNAs of NPR-B and S100A7 in mastitis goat mammary gland tissues were apparently upregulated compared to that in healthy goat (*p* < 0.01) (Figure 6C). There was no significant difference in mRNA of CNP between healthy and mastitis goat mammary gland tissues (*p* > 0.05) (Figure 6C). These results suggested that the expression of NPR-B and S100A7 was upregulated in mastitis goat mammary gland.

**Figure 7:**
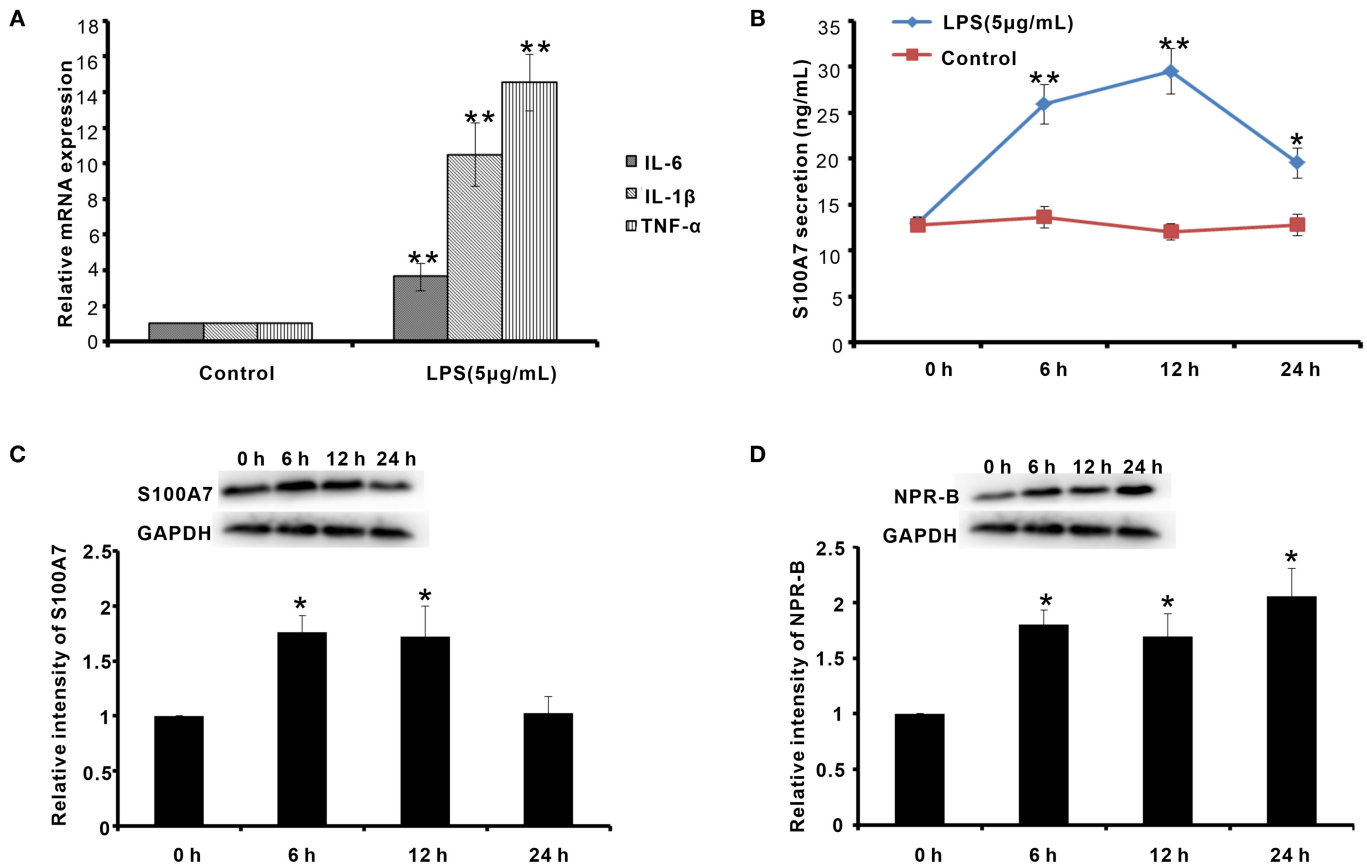
Establishment of goat mastitis model *in vitro* and the expression of NPR-B and S100A7 in goat mastitis model *in vitro*. **(A)** Quantitative PCR results showing the mRNAs expression level of interleukin-6 (IL-6), interleukin-1β (IL-1β), and tumor necrosis factor-α (TNF-α) in goat MECs treated by 5 μg/ml LPS for 6 h. **(B)** ELISA results showed the S100A7 concentration in medium in which goat MECs were cultured and treated by 5 μg/ml LPS for 0, 6, 12, and 24 h, respectively. **(C)** The Western blotting and quantitative analysis results of the S100A7 expression level in goat MECs treated by 5 μg/ml LPS for 0, 6, 12, and 24 h, respectively. **(D)** The Western blotting and quantitative analysis results of the NPR-B expression level in goat MECs treated by 5 μg/ml LPS for 0, 6, 12, and 24 h, respectively. *p < 0.05 (compared to control); **p < 0.01 (compared to control). Full-length blots are given in Supplementary Figures S19–S21.

On the other hand, the goat mastitis model was established *in vitro* using goat MECs treated by LPS. After treatment with 5 μg/ml LPS for 6 h, the mRNAs of inflammatory response-related factors, including IL-6, IL-1β, and TNF-α, were obviously increased compared to control (0 μg/ml LPS) (*p* < 0.01) (Figure 7A). Meanwhile, after treatment with 5 μg/ml LPS for 6 h, the secretion content of S100A7 was higher than that of control (0 μg/ml LPS) (*p* < 0.01) (Figure 7B). This increase in S100A7 secretion was consistent until LPS treatment for 12 h. After 24 h of treatment, the secretion of S100A7 began to decrease, but it was still higher than that of the control group (0 μg/ml LPS) (*p* < 0.05) (Figure 7B). For the protein expression of S100A7, it was higher than that of control (0 μg/ml LPS) (*p* < 0.05) after treatment with 5 μg/ml LPS for 6 and 12 h, respectively, but there was no significant difference between the 24-h group and control (0 μg/ml LPS) (*p* > 0.05) (Figure 7C). These results indicated that LPS could induce the establishment of mastitis model *in vitro* and induce the expression and secretion of S100A7 in goat MECs. Further, in this goat mastitis model *in vitro*, the expression level of NPR-B was obviously upregulated. After treatment with 5 μg/ml LPS for 6, 12, and 24 h, respectively, the expression levels of NPR-B in all of these three groups were markedly higher than that of the control group (0 μg/ml LPS) (*p* < 0.05) (Figure 7D). These results suggested that the increase in expression of NPR-B was involved in goat mastitis.

## Discussion

In goat, CNP and NPR-B were only reported mainly expressed in lung, heart, ovary, and uterus (31, 32). The expression of CNP and NPR-B was first analyzed in healthy goat mammary gland. Immunohistochemical results showed that there was only NPR-B, but not CNP positive immunoreactivity in the alveolus epithelial cells. In order to confirm this result, goat MECs were isolated and detected the expression of natriuretic peptides family and their receptors. The results of RT-PCR confirmed the immunohistochemistry results that the goat MECs, only expressed the mRNA of NPR-B, did not express the mRNA of NPPC. In this study, NPR-B immunoreactivity was observed inside of the cells, rather than the cell membrane. Although NPR immunoreactivity should be observed on the cell membrane in theory, many articles have shown that NPR immunoreactivity sometimes could be observed inside of cells (33–35). This phenomenon may be caused by the step of membrane permeability in the process of immunohistochemistry or immunofluorescence. There are few reports about the expression of natriuretic peptides family and their receptors in MECs. Previous receptor autoradiography studies were suggestive of the existence of NPR-B in goat mammary gland (36). In this study, the results of mRNA and protein level results provided direct evidence for the expression of NPR-B in goat MECs. CNP has the highest affinity with NPR-B (37). But, the goat MECs expressed BNP rather than CNP. The highest affinitive receptor for BNP is NPR-A, which was not expressed in goat MECs.

Since goat MECs expressed NPR-B, the role of NPR-B in the physiological function of MECs is unclear. Interestingly, the expression and secretion of antimicrobial peptide S100A7 in goat MECs were obviously induced by CNP/NPR-B signaling pathway. Since goat MECs treated by CNP alone, the protein expression level and the secretion content of S100A7 significantly increased compared with control. S100A7 is considered as a critical component of the animal innate immunity system (38). Many inflammatory cytokines such as IL-6, IL-17, IL-22, and TNF-α could upregulate the expression of S100A7 (24, 25). The inductive effect of CNP on the expression and secretion of S100A7 was the first report in this study, leading to a hypothesis that CNP/NPR-B signaling pathway may take part in innate immunity and local mammary gland defense by regulating the expression and secretion of S100A7. In order to test and verify this hypothesis, the activation of CNP/NPR-B signaling pathway was first detected. NPR-B is a guanylate cyclase coupled receptor. Its physiological function depends on the guanylate cyclase activity. As predicted, after treatment with CNP, the cGMP level in goat MECs was significantly upregulated. The main cGMP effector is protein kinase G (PKG) and its targets include JNK (39, 40). The expression of S100A7 is regulated by an AP-1-responsive promoter (26). c-Jun, which is an important subunit of AP-1, is the phosphorylated target of JNK. Along with the upregulation of cGMP level, the phosphorylation levels of JNK and its target c-Jun also increased gradually after CNP treatment. These results indicated that CNP could activate NPR-B/JNK/c-Jun signaling pathway in goat MECs. Second, whether the inductive effect of CNP on the expression and secretion of S100A7 was dependent on NPR-B/JNK/c-Jun signaling pathway? KT5823 is a specific inhibitor for PKG. As shown in Figure 5, KT5823 remarkably inhibited the phosphorylation of JNK and c-Jun induced by CNP. Correspondingly, KT5823 evidently inhibited the expression and secretion of S100A7 induced by CNP.

Mastitis is an inflammatory reaction of udder tissue commonly caused by bacterial infection (41). CNP/NPR-B, which induced goat MECs to express and secrete S100A7, may be involved in the occurrence or prevention of mastitis in goat. The expression of CNP, NPR-B, and S100A7 in mammary gland tissue from healthy and mastitis goats was analyzed. The results suggested that the expression of NPR-B and S100A7 was upregulated in mastitis goat mammary gland. But, there was no significant difference in expression of CNP between healthy and mastitis goat mammary gland tissues. On the other hand, goat mastitis model was established *in vitro* using goat MECs treated by LPS. LPS, which is a cell wall component of Gram-negative bacteria, was widely used to induce mastitis model *in vivo* and *in vitro* (42–45). It is reported that LPS from the commensal microbiota of the human breast induced the expression of S100A7 in breast cancer cells (46). Consistent with previous reports, after treatment with LPS, the inflammatory response-related factors, including IL-6, IL-1β, and TNF-α, were obviously increased. Moreover, LPS could induce the expression and secretion of S100A7 in goat MECs. Similar to the results *in vivo*, in goat mastitis model *in vitro*, the expression level of NPR-B was obviously upregulated.

Based on these results, we speculate that CNP/NPR2 through inducing the expression and secretion of S100A7 could play an important role in innate immunity and local mammary gland defense. However, the goat MECs did not express CNP. There may be two sources of CNP in goat mammary gland. First is adipose tissue in udder. Recently, many reports suggested that adipose tissue could express CNP (13, 47). Cumulating evidence indicates that the communication between mammary gland and adipose tissue plays a critical role in mammary gland remodeling (48–50). Therefore, the interaction between mammary gland and adipose tissue in udder innate immunity may be the next study focus. Second is inflammatory cells infiltrated into alveolus from blood. During mastitis, inflammatory cells such as monocytes, neutrophils, T cells, and macrophages invade into alveolus and expressed CNP (51, 52) to induce the expression and secretion of S100A7 from MECs. This possibility also needs further study (53, 54).

**Figure 8:**
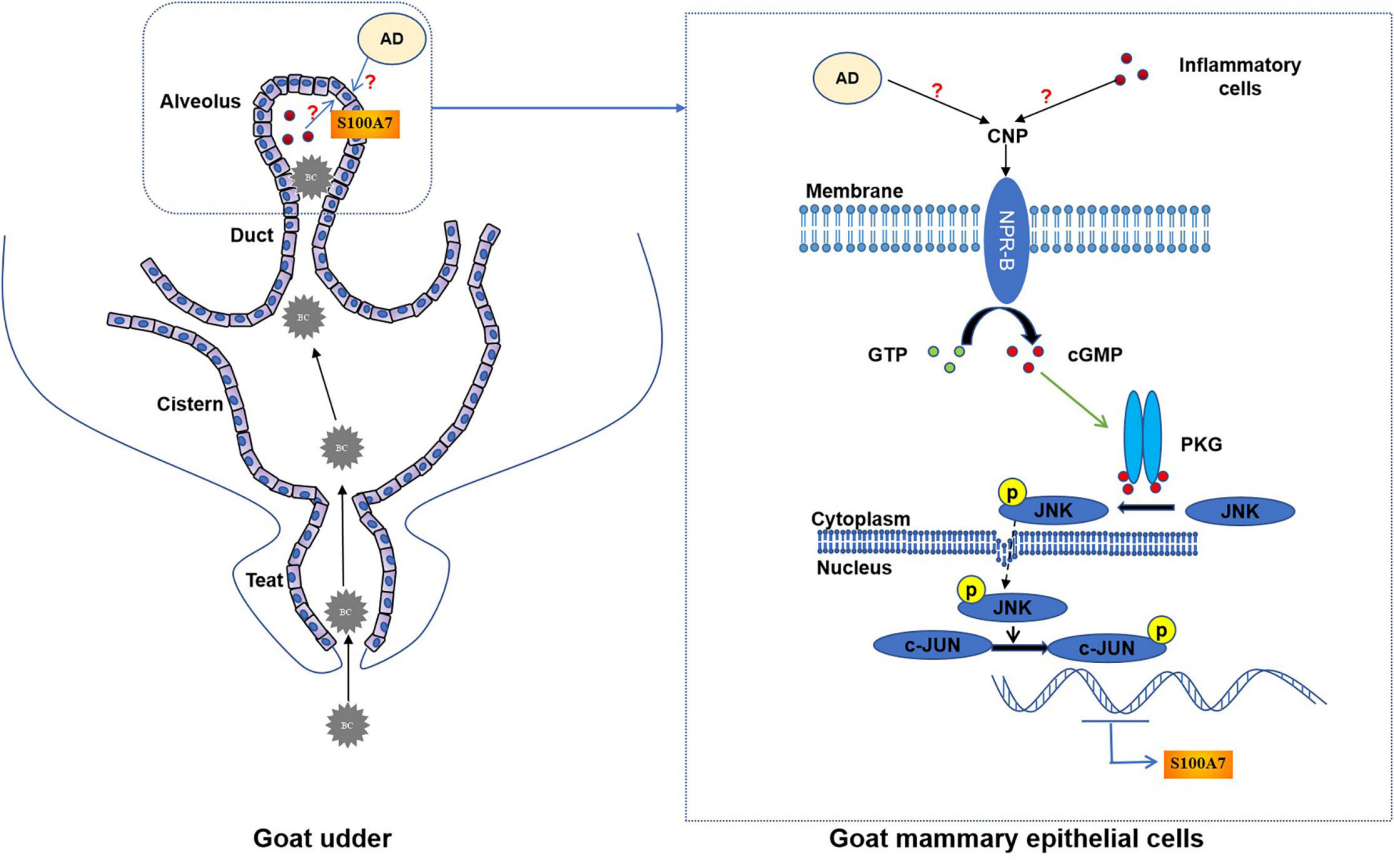
The schematic mechanistic diagram. In goat udder, bacteria (BC) pass through the teat canal and then enter the cistern and alveolus, resulting in intramammary infection. During intramammary infection, adipose tissue (AD) or inflammatory cells may secrete CNP. CNP binds to NPR-B and activates the guanylate cyclase activity of NPR-B. Activated NPR-B induces the production of cyclic guanosine monophosphate (cGMP), thereby activating PKG. PKG phosphorylates JNK and phosphorylated JNK phosphorylates the downstream target c-Jun. Finally, phosphorylated c-Jun as a transcription factor induced the expression of S100A7 in MECs.

In conclusion, goat mammary gland expressed NPR-B, indicating mammary gland may be a target organ for the natriuretic peptide system. Moreover, CNP, through NPR-B/JNK/c-Jun signaling pathway to regulate the expression and secretion of S100A7 in MECs, plays an important role in host mammary gland innate immunity (Figure 8).

## Data Availability Statement

The datasets presented in this study can be found in online repositories. The names of the repository/repositories and accession number(s) can be found in the article/Supplementary Material.

## Ethics Statement

The animal study was reviewed and approved by the Institutional Animal Ethical and Welfare Committee, Northwest A&F University, Shaanxi, China.

## Author Contributions

MF, YM, and QW designed the study. MF and YM performed the majority of the experiments. YY, KZ, and XZ contributed to the regents and materials. MF, QW, and MP analyzed the data. MF, QW, and BM wrote the manuscript. All authors contributed to the article and approved the submitted version of the manuscript.

## Funding

This study was supported by the National Natural Science Foundation of China (31772818).

## Conflict of Interest

The authors declare that the research was conducted in the absence of any commercial or financial relationships that could be construed as a potential conflict of interest.

## Publisher's Note

All claims expressed in this article are solely those of the authors and do not necessarily represent those of their affiliated organizations, or those of the publisher, the editors and the reviewers. Any product that may be evaluated in this article, or claim that may be made by its manufacturer, is not guaranteed or endorsed by the publisher.
