# Supplementary material for: C-Type Natriuretic Peptide Regulates the Expression and Secretion of Antibacterial Peptide S100A7 in Goat Mammary Gland Through PKG/JNK/c-Jun Signaling Pathway
Source: Front Vet Sci. 2022 Apr 12;9:822165. doi: 10.3389/fvets.2022.822165 (PMC9039262; doi:10.3389/fvets.2022.822165)
Supplement: Supplementary file 1 [file Data_Sheet_1.DOCX]

Supplementary Material

# Material and methods

**Oil Red O staine**

After treatment with CNP, cells were fixed in 4% paraformaldehyde for 20 min in room temperature, Oil Red O staining was performed. The staining fluid was removed, followed by wash three times with PBS. Isopropyl alcohol was added to dissolve lipid drops, and the absorbance was measured at 550 nm using a microplate reader (Invitrogen Corporation, Waltham, MA).

**Cell counting kit 8 assay for cell viability**

Cell viability assays were performed using cell counting kit 8. In short, after detaching with 0.25% trypsin, the cells were cultured in 96-well microtiter plates in a final volume of 100 μL culture medium per well at the density of 5 × 10^3^ cells in each well. Cells were incubated with different concentrations of CNP, and there were six replicates in each group (n = 6). After the cells were exposed to CNP for corresponding days, the medium in each well was replaced with 100 μL fresh DMEM/F12 medium and 10 μL of CCK reagent. Then the 96-well plates were in foil to avoid light and incubated for a further 2 s. The absorbance at 450 nm was measured using the Model 3550 microplate reader (Bio-Rad, California).

**Cell cycle assay**

Cells (3 × 10^5^) were seeded into each well of a 6-well plate and treated with CNP for 72 h. Then the cells were harvested, fixed with 70% cold ethanol at 4 °C overnight and washed with PBS. The cells were then stained with propidium iodide (PI) with RNaseA (100 μg/ml) for 30 min in the dark and washed with PBS, and the cell cycle distribution was assessed by using a FACS Aria flow cytometer (BD Biosciences, San Jose, CA, USA) at 488 nm.

# Supplementary Figures

**Supplementary Figure S1: CNP treatment did not effect on synthesis of β-casein and fatty acid in goat MECs.**  **(A)**, The mRNA expression level of β-casein, ACACA and SREBF1 detected by quantitative PCR after CNP or prolactin treatment for 24 h. **(B)**, Oil Red O stained results in goat MECs after CNP treatment for 24 h, 48 h. *: p < 0.05(compared to control); **: p < 0.01 (compared to control).

**Supplementary Figure S2: CNP treatment did not effect on cell proliferation of goat MECs. (A)**, Cell counting kit 8 assay results in goat MECs after treatment with CNP. **(B)**, Cell cycle assay in goat MECs after treatment with CNP for 72 h.


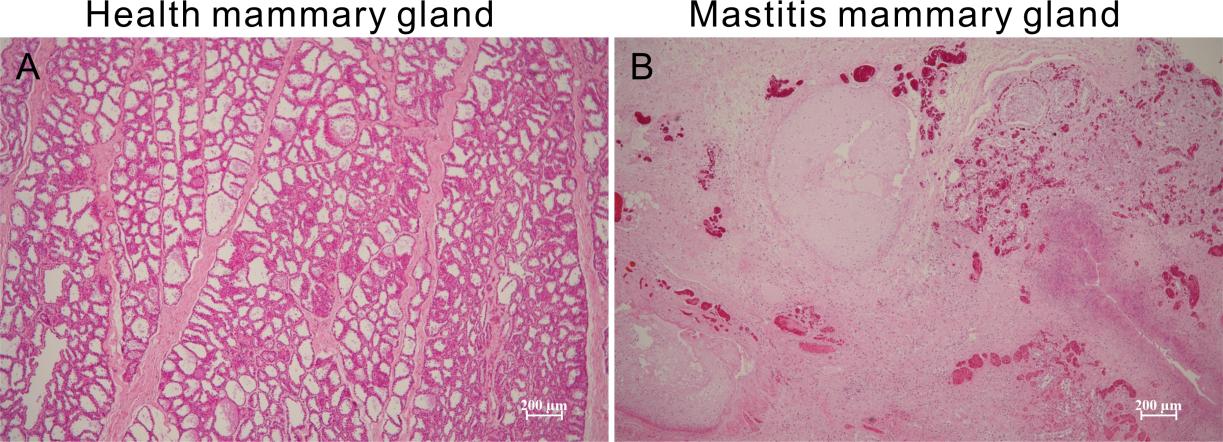


**Supplementary Figure S3: Representative images of Hematoxylin-Eosin staining in health and mastitis goat mammary gland tissues. (A)**, Health goat mammary gland tissue; **(B)**, Mastitis goat mammary gland tissue. Bar=200μm.


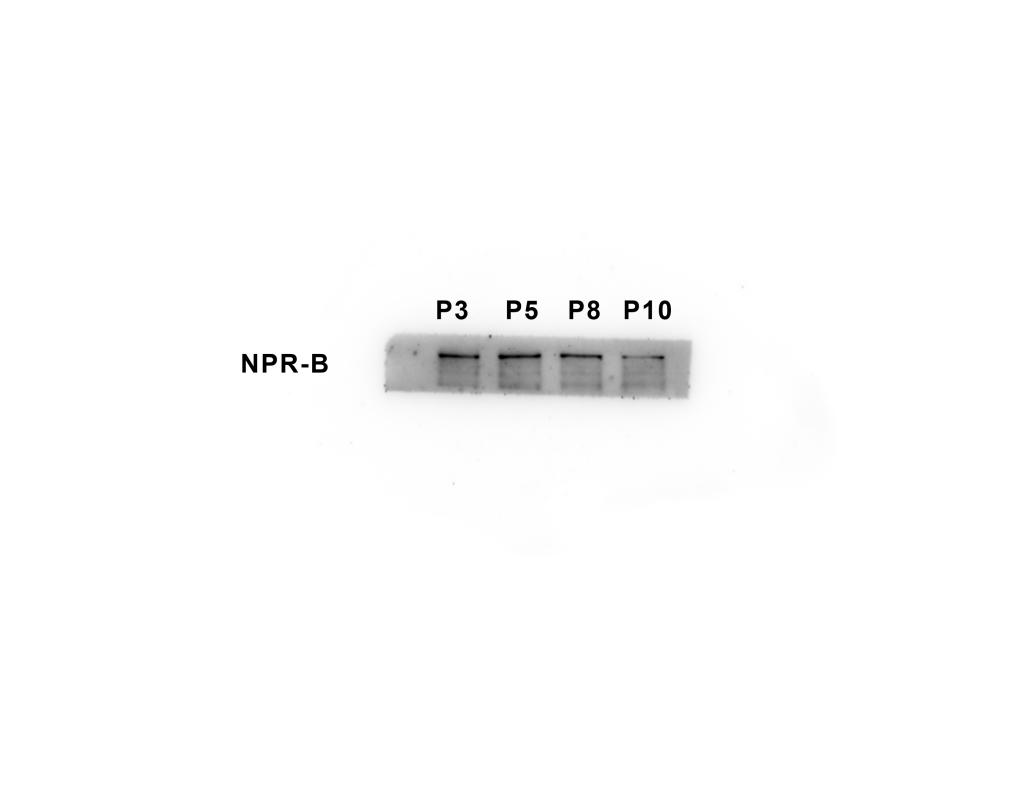


**Supplementary figure S 4. Orignial image for Fig 2. F NPR-B**


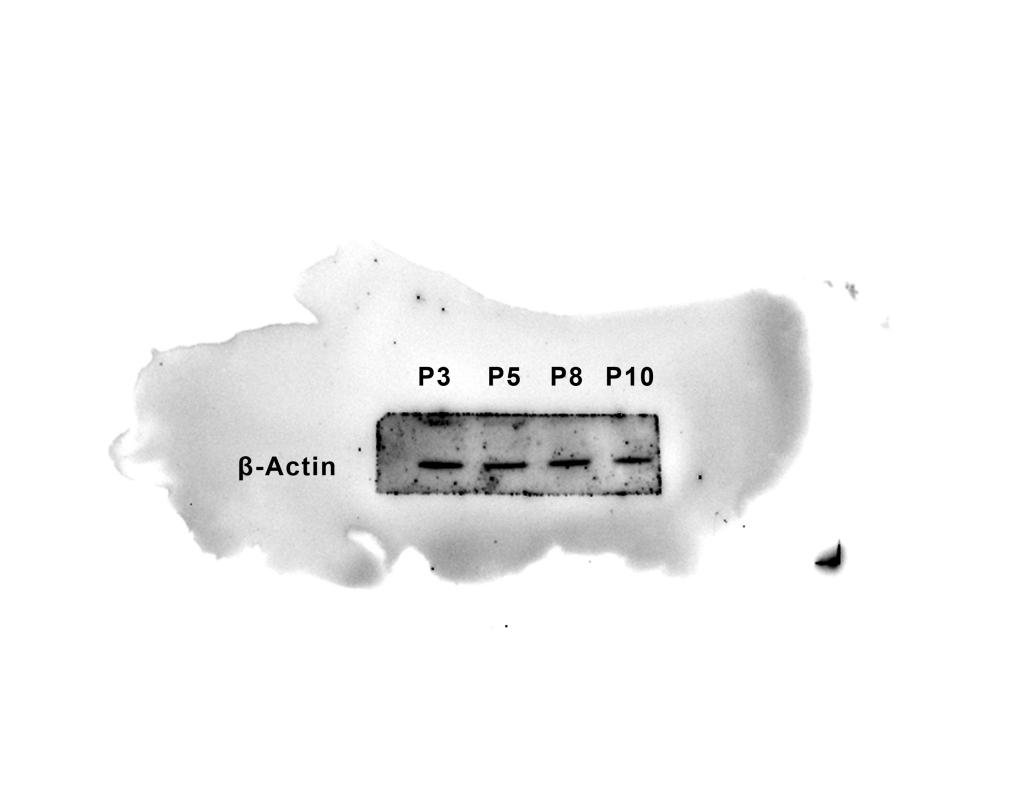


**Supplementary figure S 5. Orignial image for Fig 2. F actin**

**
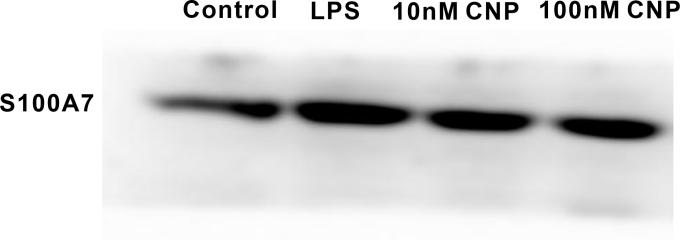
**

**Supplementary figure S 6. Orignial image for Fig 3. A S100A7**

**
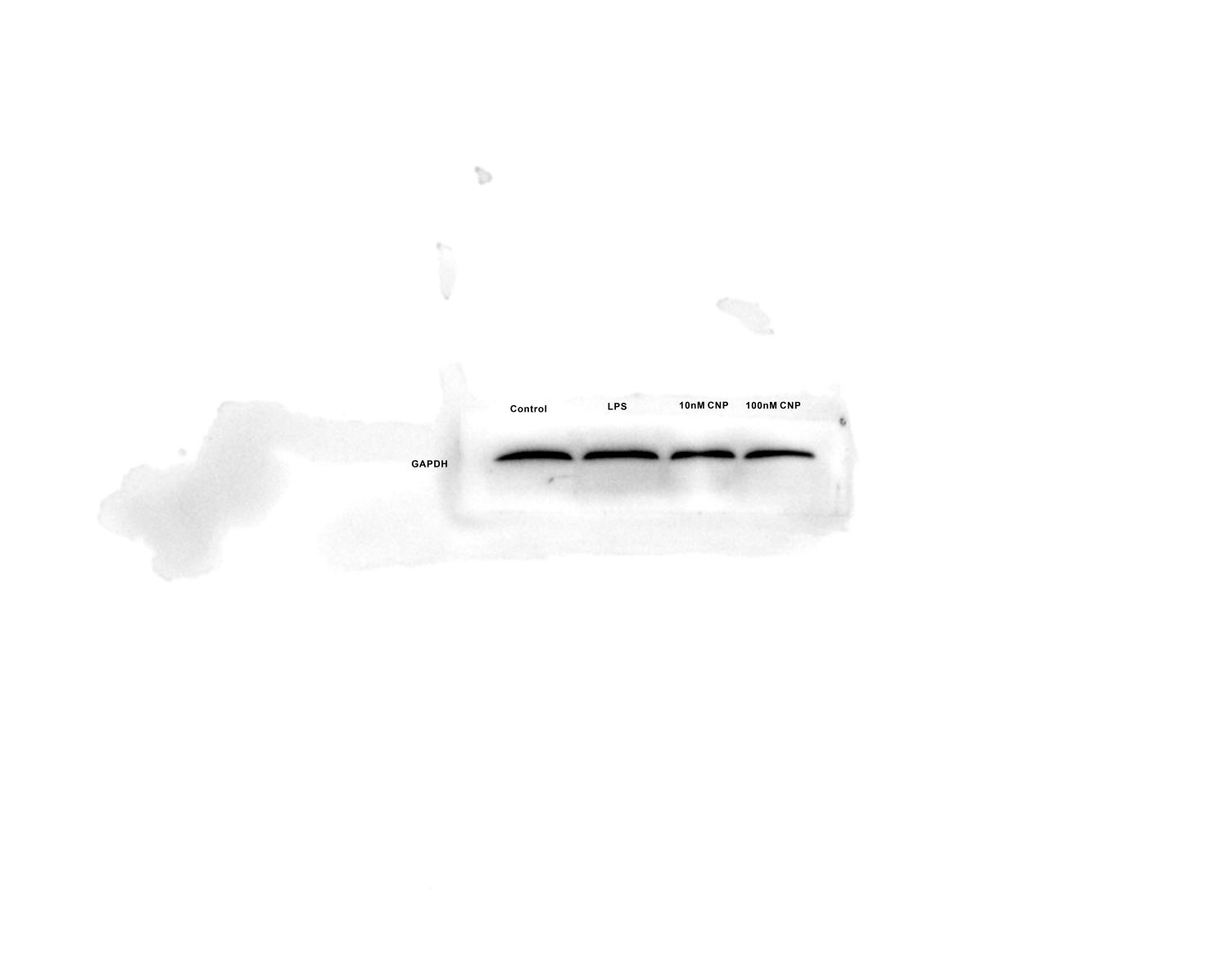
**

**Supplementary figure S 7. Orignial image for Fig 3. A GAPDH**


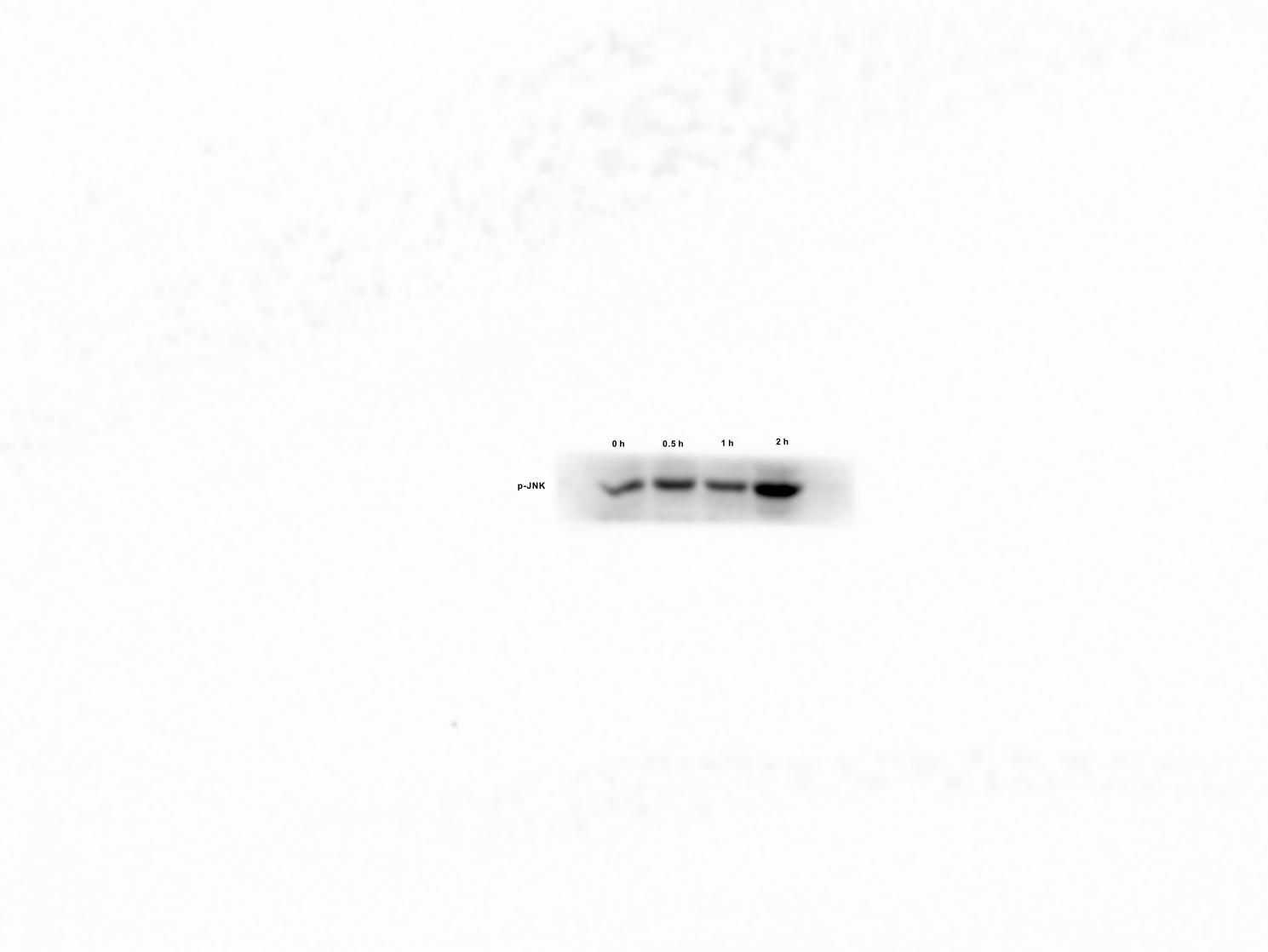


**Supplementary figure S 8. Orignial image for Fig 4. B p-JNK**


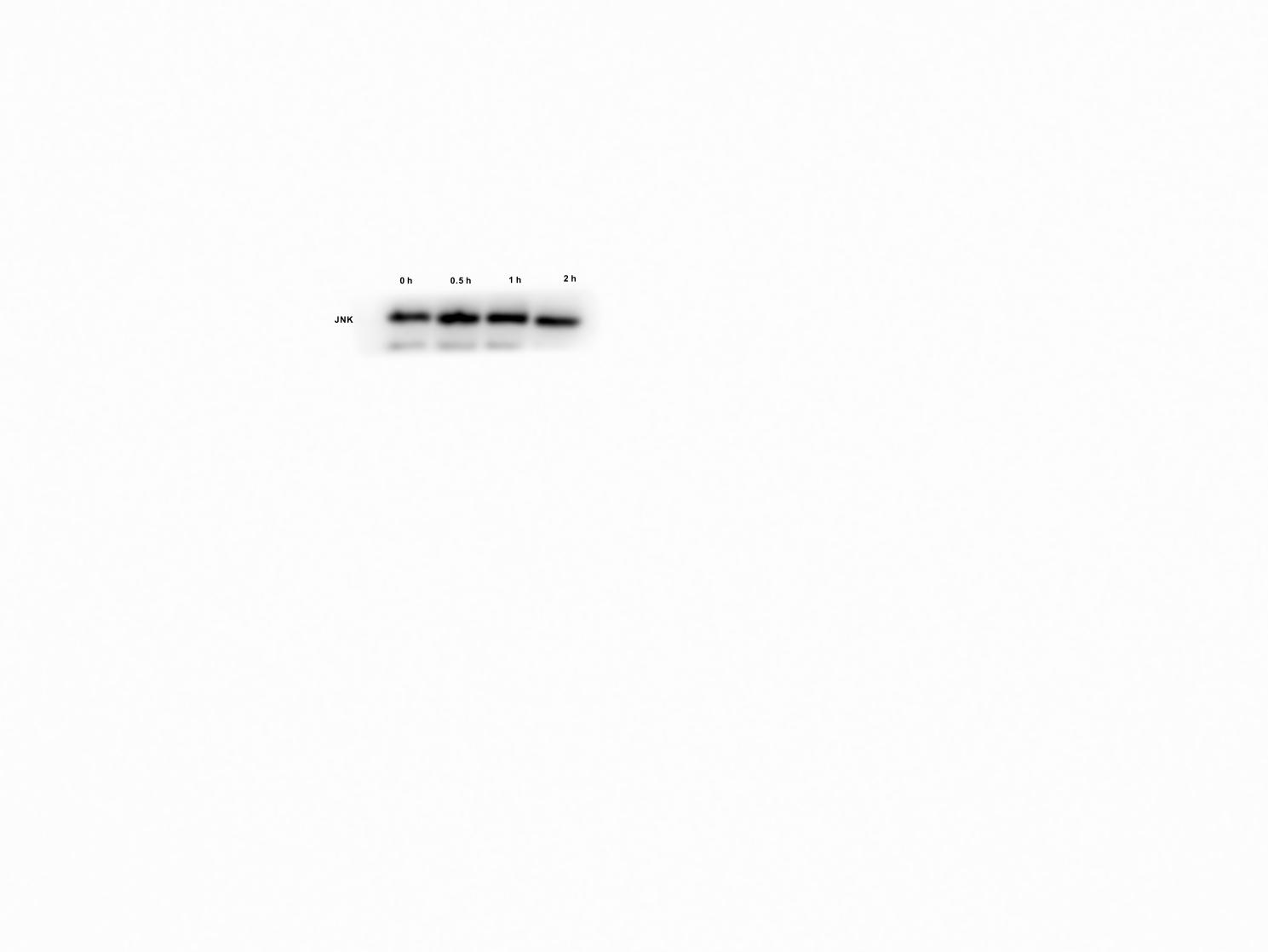


**Supplementary figure S 9. Orignial image for Fig 4. B JNK**


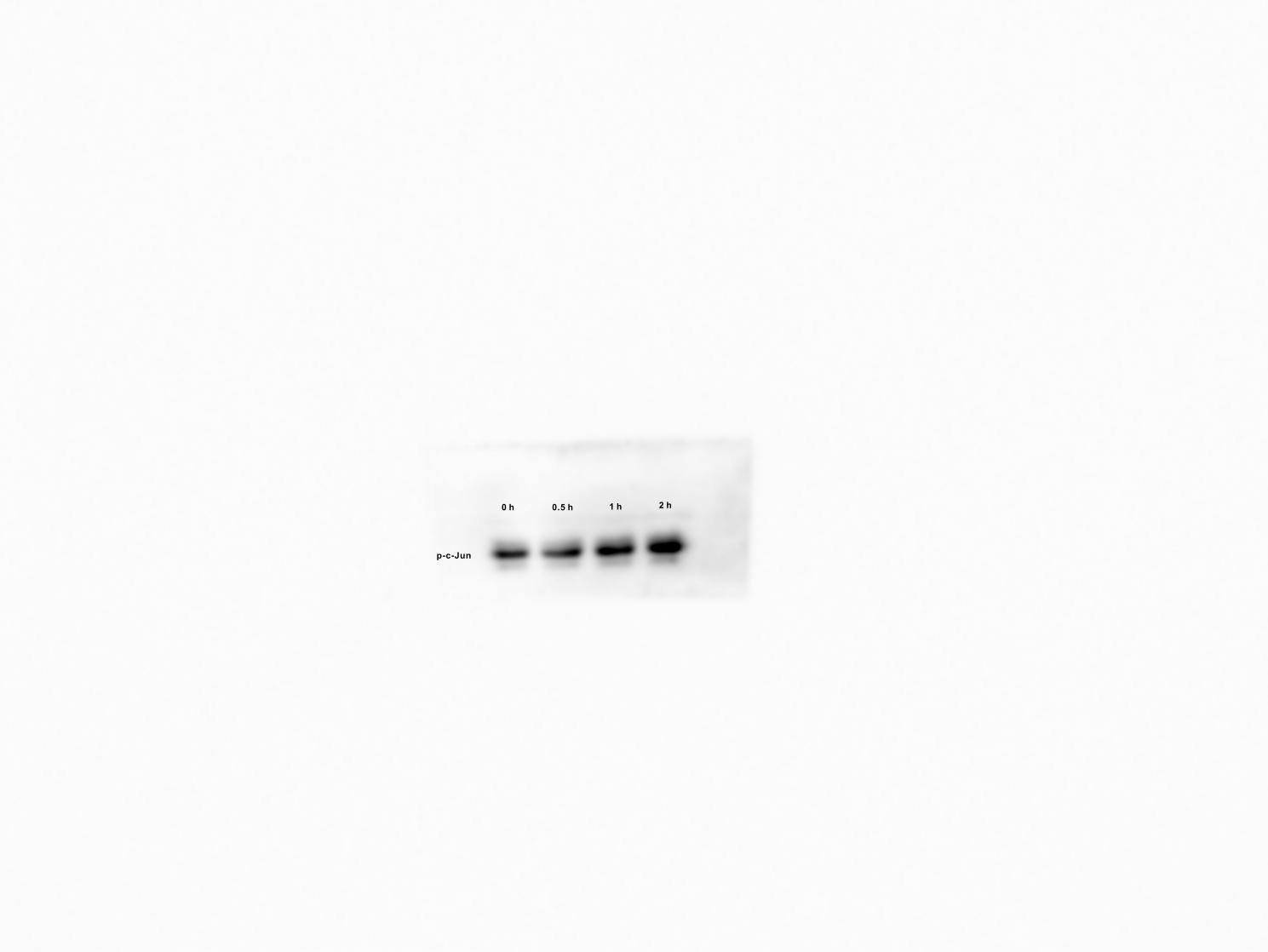


**Supplementary figure S 10. Orignial image forFig 4. B p-c-Jun**

**
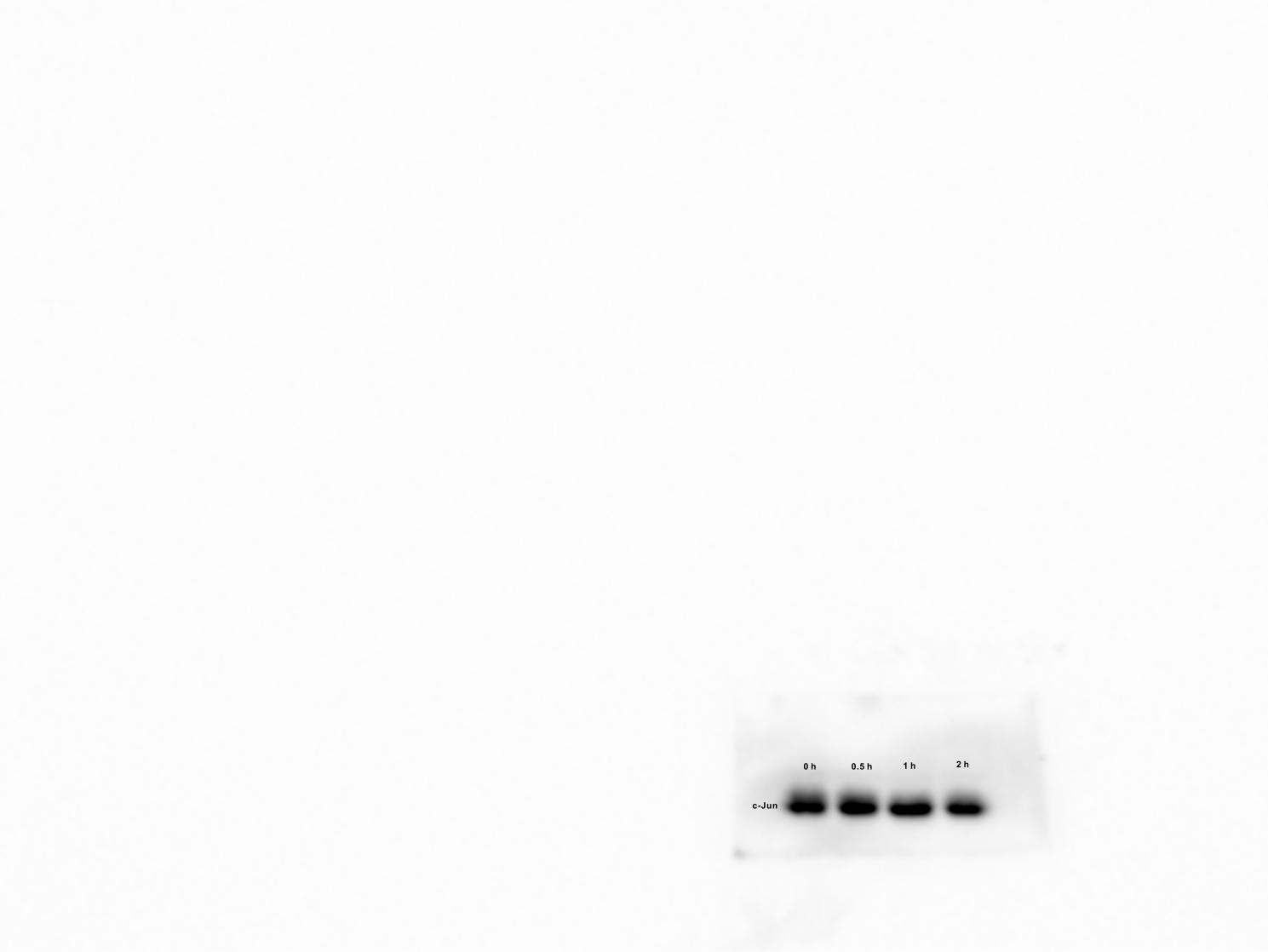
**

**Supplementary figure S 11. Orignial image for Fig 4. B c-Jun**

**
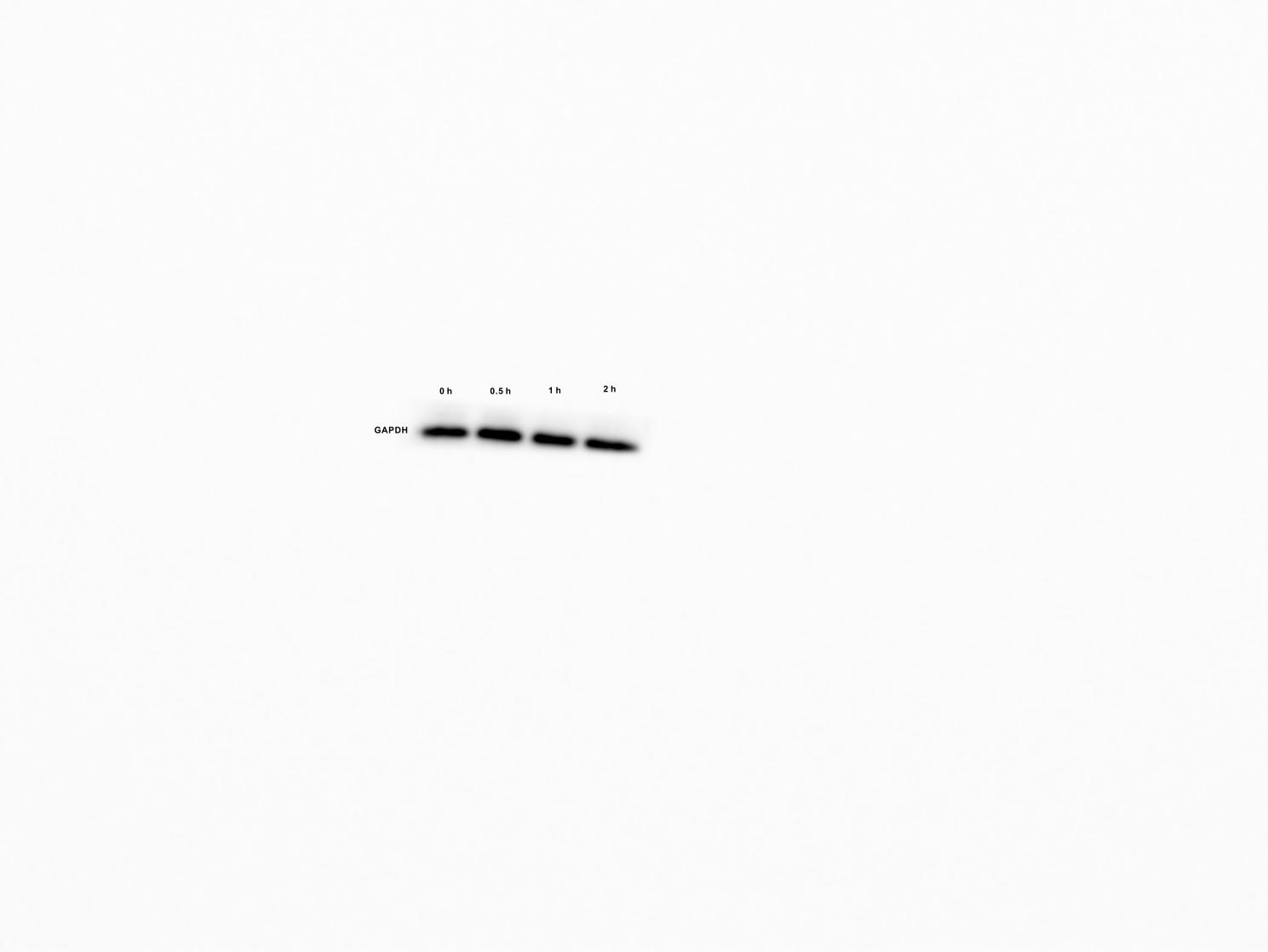
**

**Supplementary figure S 12. Orignial image for Fig 4. B GAPDH**


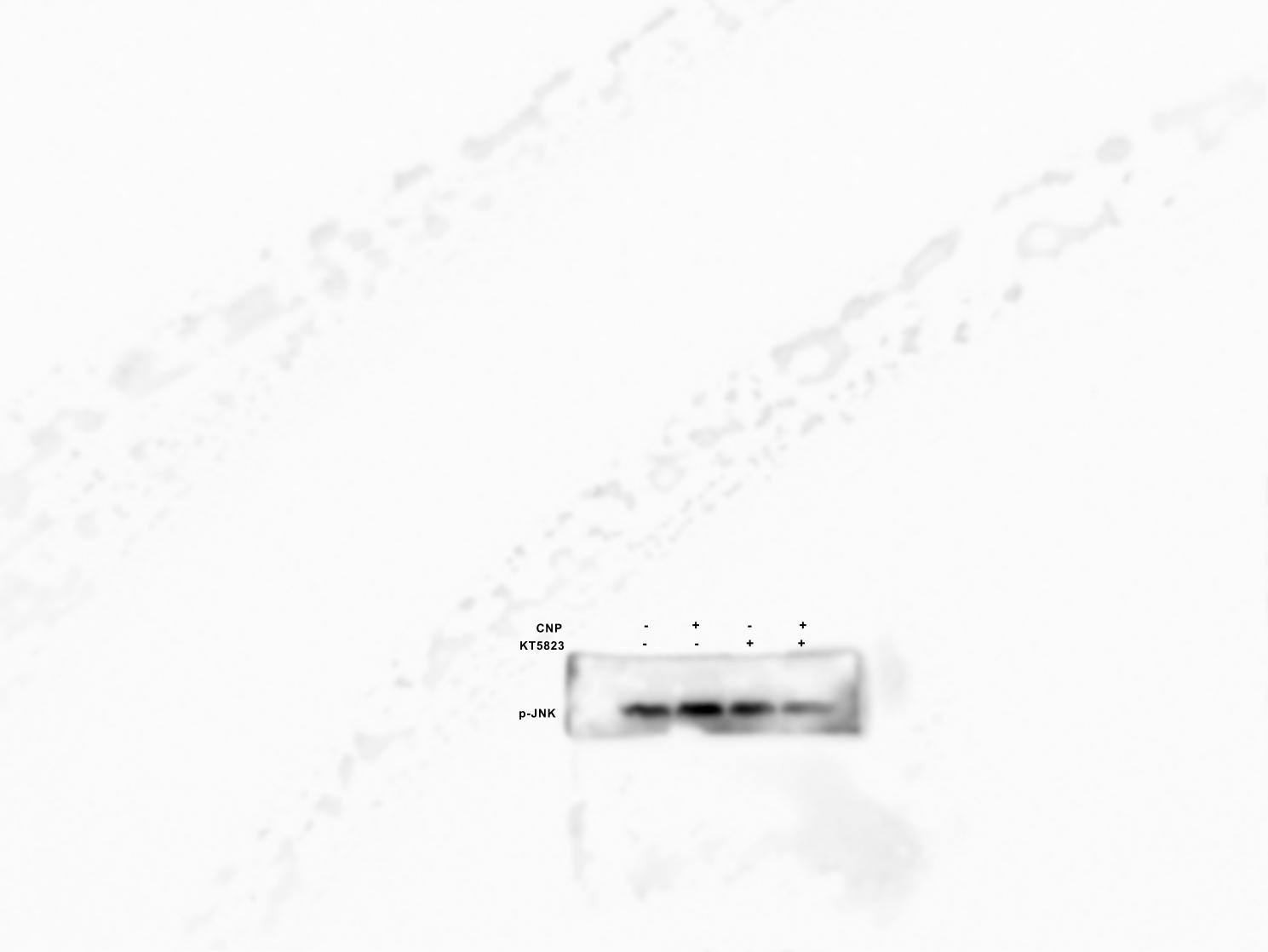


**Supplementary figure S 13. Orignial image for Fig 5. A p-JNK**

**
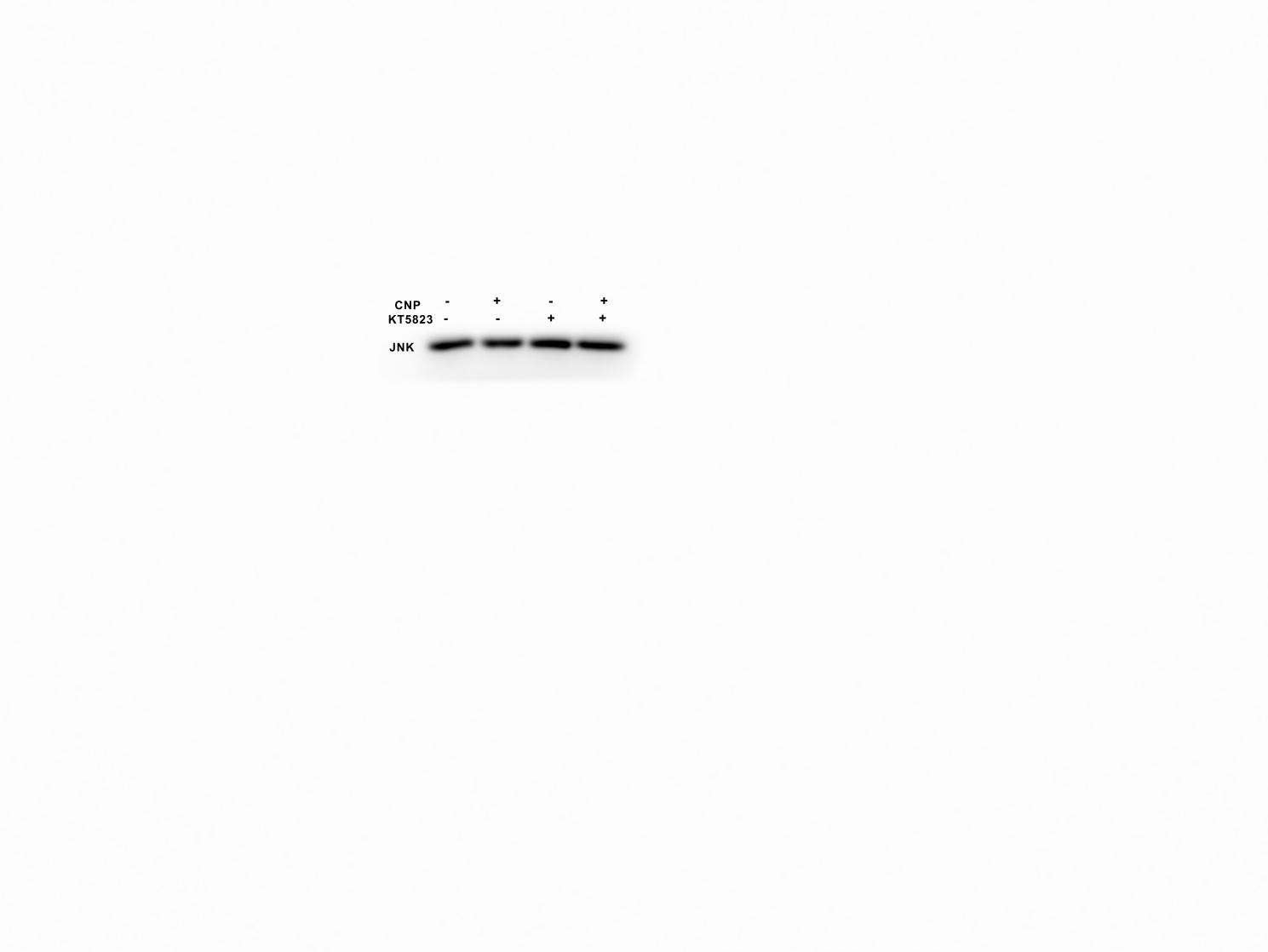
**

**Supplementary figure S 14. Orignial image for Fig 5. A JNK**


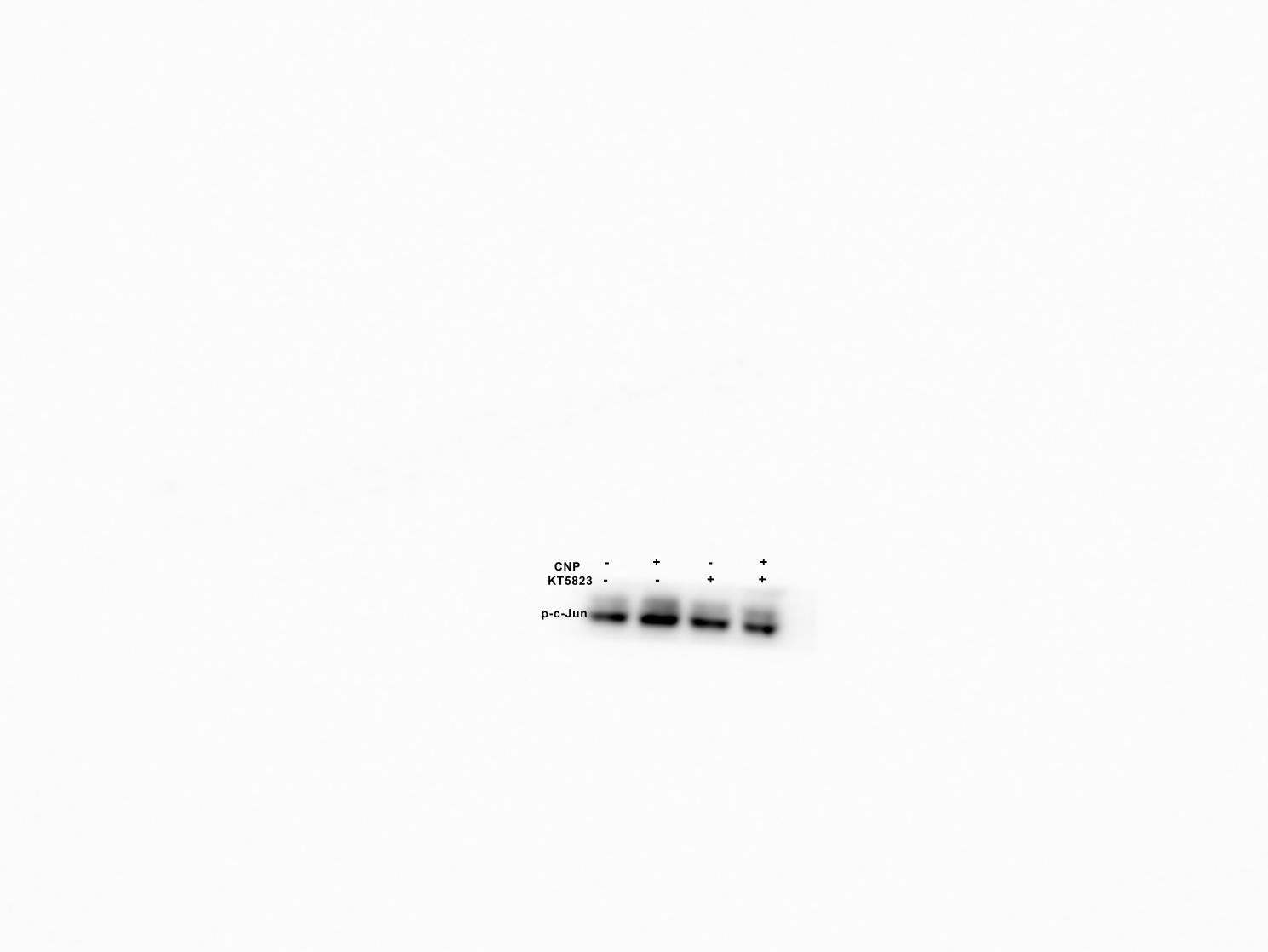


**Supplementary figure S 15. Orignial image for Fig 5. A p-c-Jun**


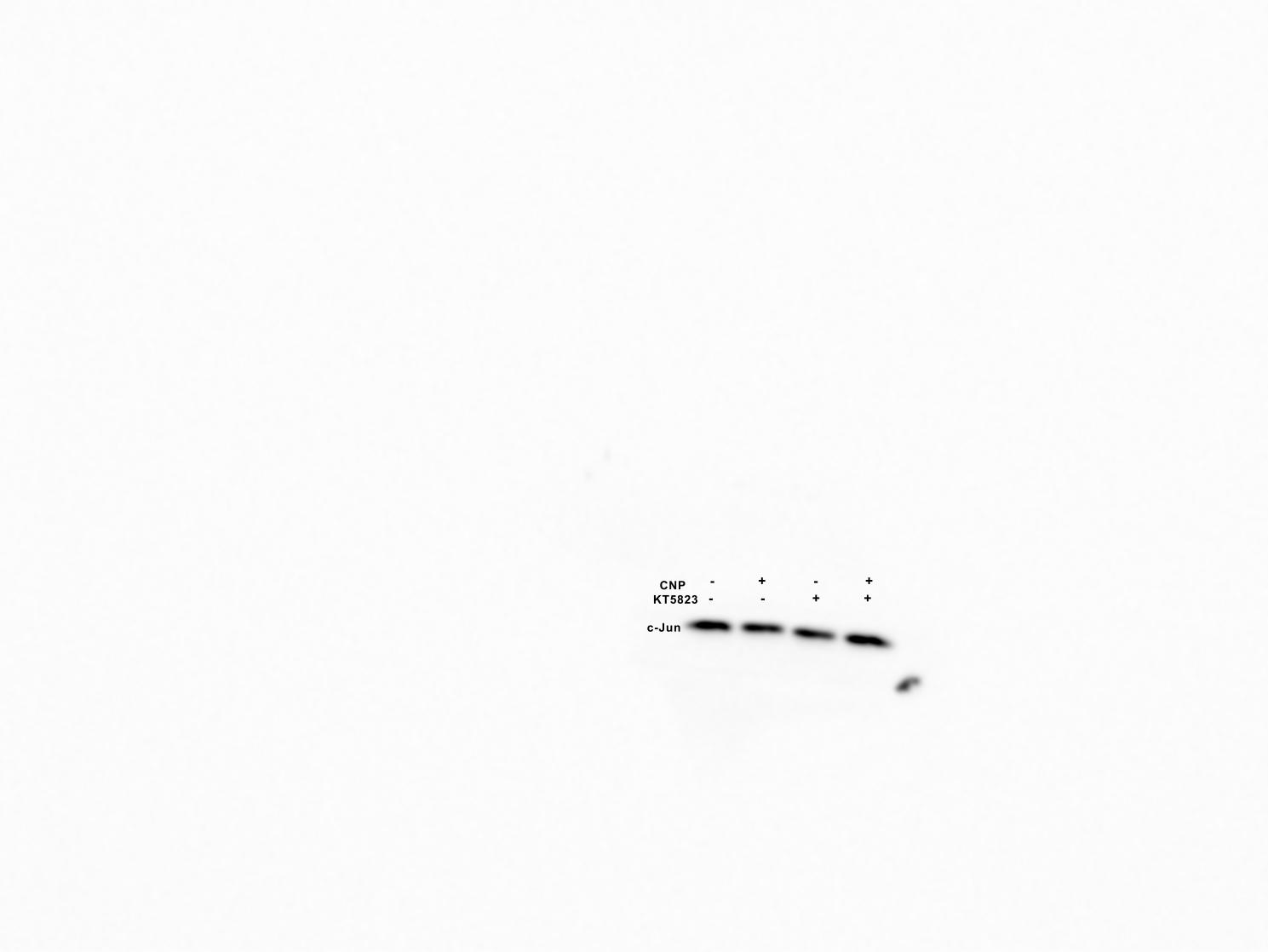


**Supplementary figure S 16. Orignial image for Fig 5. A c-Jun**


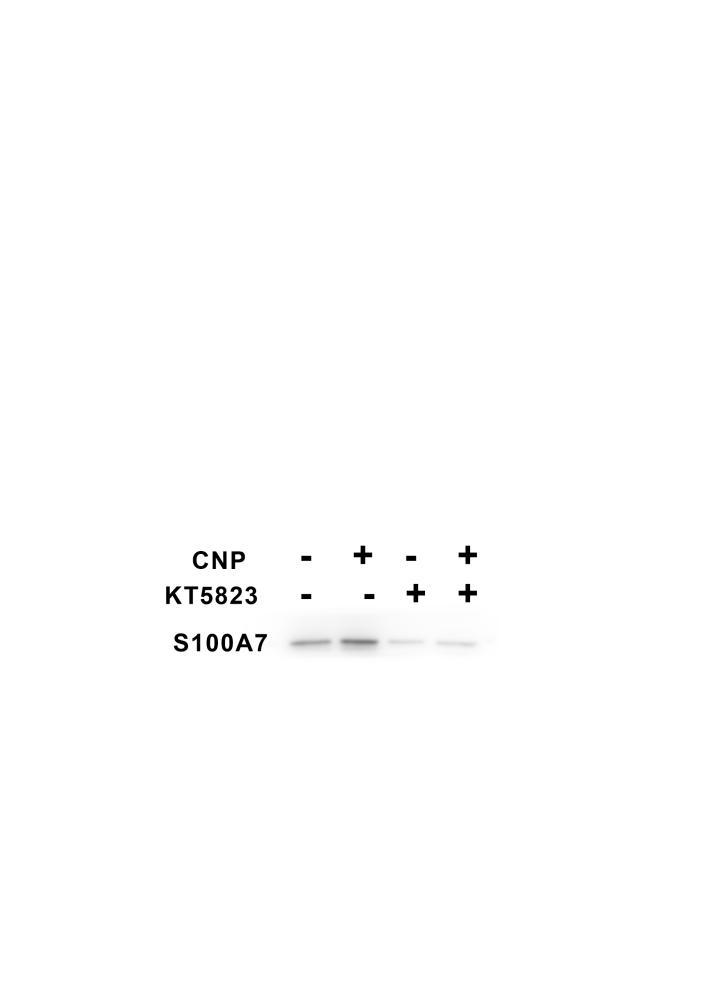


**Supplementary figure S 17. Orignial image for Fig 5. B S100A7**

**
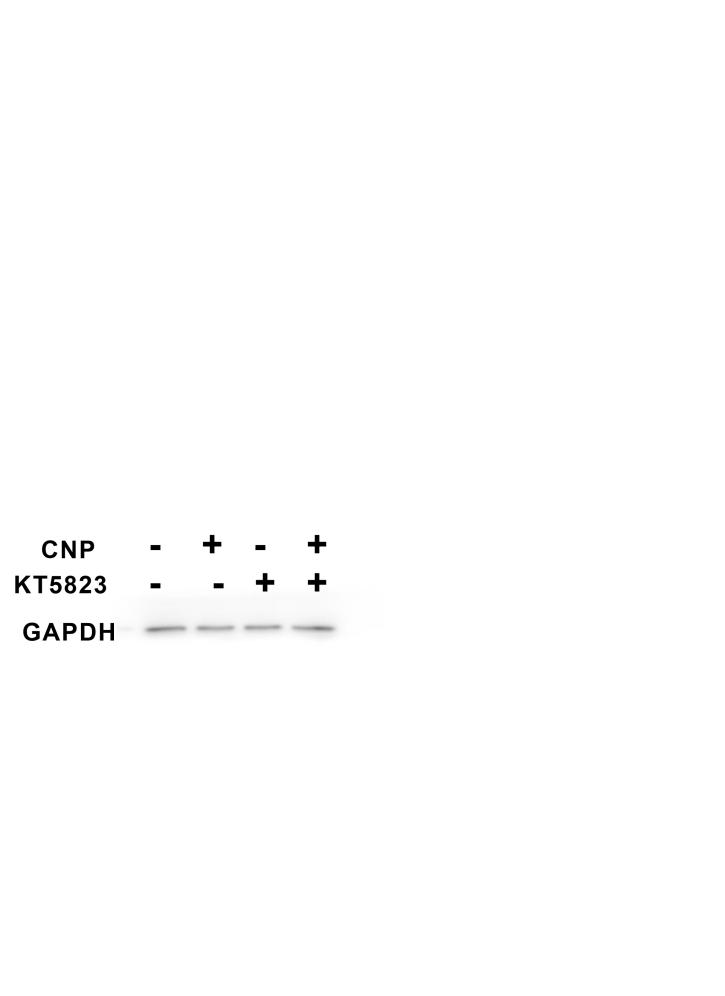
**

**Supplementary figure S 18. Orignial image for Fig 5. B GAPDH**

**
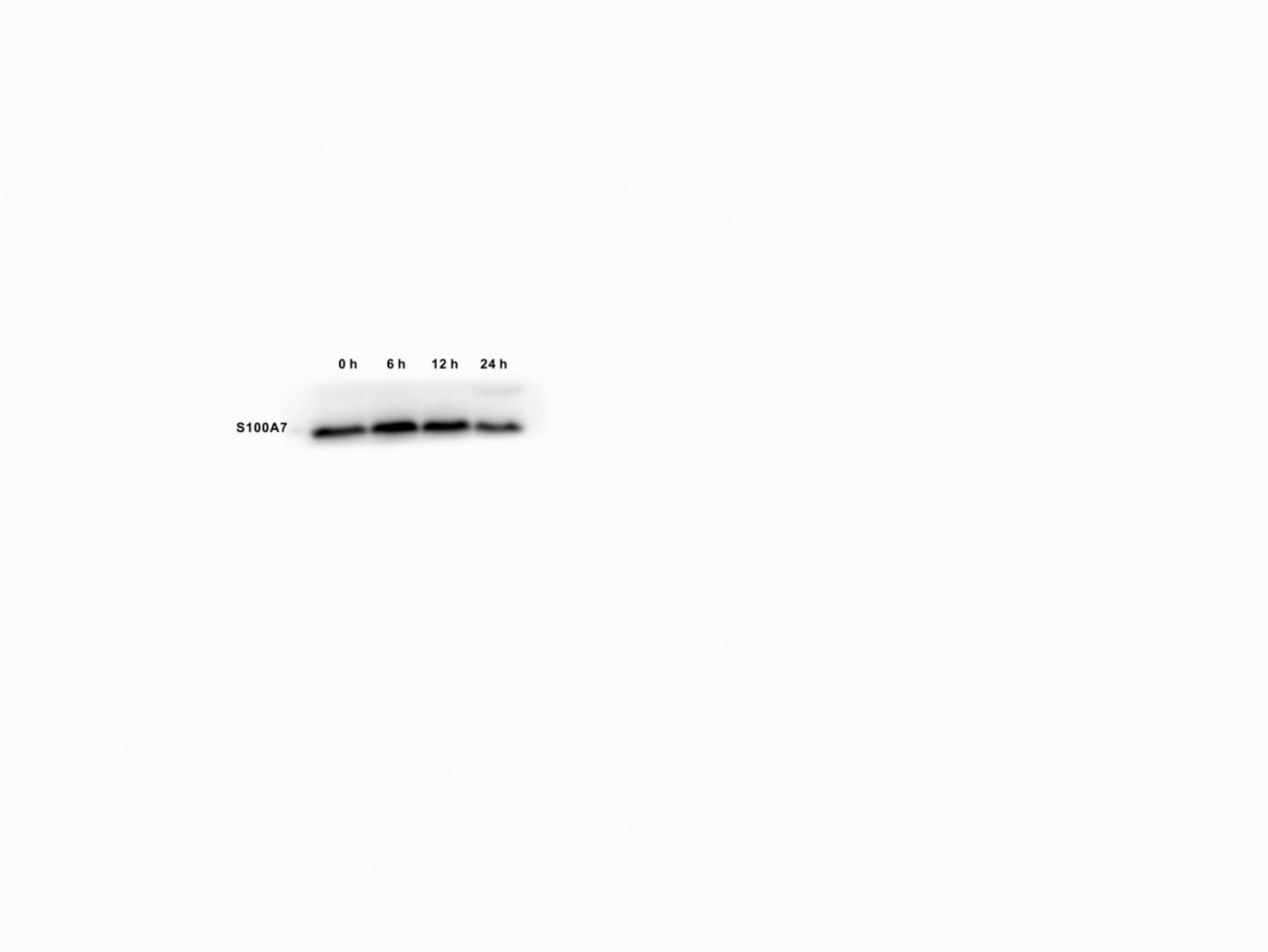
**

**Supplementary figure S 19. Orignial image for Fig 7. C S100A7**

**
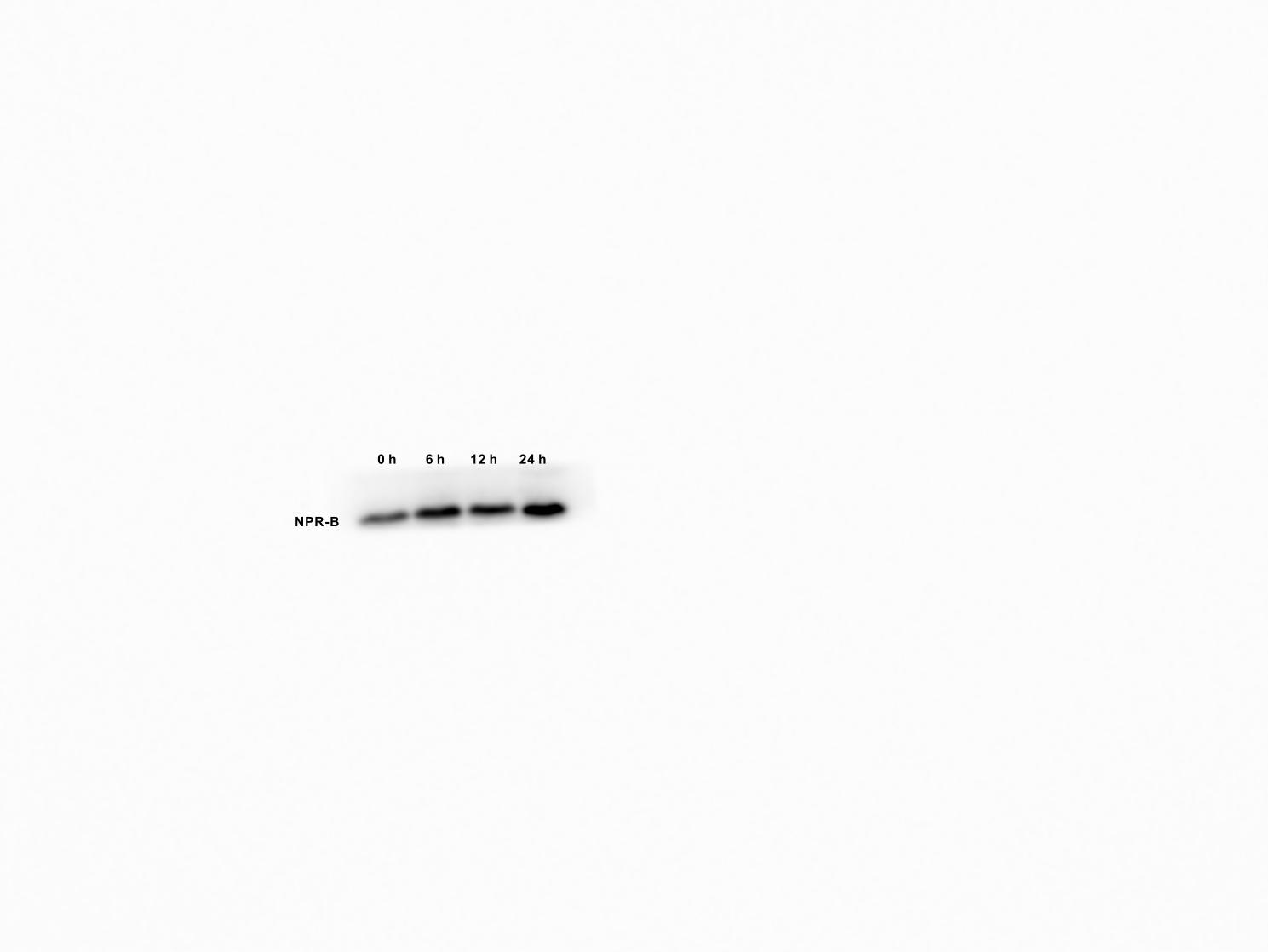
**

**Supplementary figure S 20. Orignial image for Fig 7. D NPR-B**

**
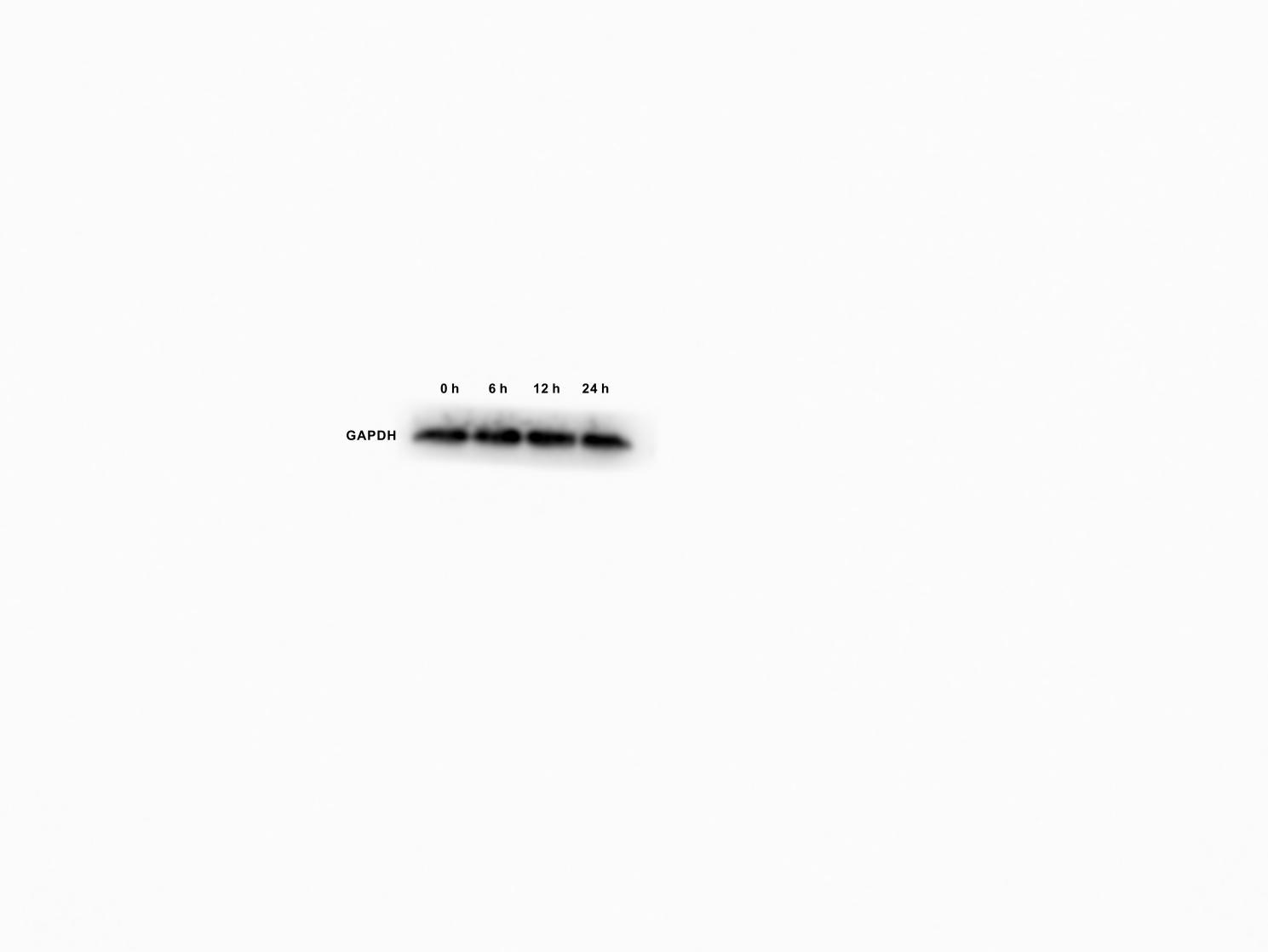
**

**Supplementary figure S 21. Orignial image for Fig 7. C, D GAPDH**

# Supplementary Tables

# **Supplementary table S1: Primer sequences for the RT-PCR and q-PCR analyses**

| Gene | Primer sequences (5’ → 3’) | Product size, bp | Anneal T, °C | Genbank accession |
| --- | --- | --- | --- | --- |
| GAPDH | F:TGCCCGTTCGACAGATAGC | 145 | 60 | XM_005680968.3 |
|  | R: ACGATGTCCACTTTGCCAGTA |  |  |  |
| S100A7 | F:CCAGCAAGGACAGGAACTCA | 140 | 60 | XM_005677510.2 |
|  | R: GCAGCTGCTGAAGGAGAACT |  |  |  |
| NPPA | F:CCTCTCAGCCCTCTTTCAGA | 202 | 60 | NM_001287236.1 |
|  | R:CTCCAATCCTGTCCATCCTTC |  |  |  |
| NPPB | F:GCTTCTCCTCTTCTTGCACCT | 93 | 60 | XM_018059984.1 |
|  | R:TCCTGTAACCCAGGCAGTTC |  |  |  |
| NPPC | F:AAGAAGGGCGACAAGACTCC | 177 | 60 | NM_001285679.1 |
|  | R:GCCCTTGGACAAACCCTTCT |  |  |  |
| NPR-A | F:GCTTCCAAGGTGTGACAGGA | 98 | 60 | XM_018046143.1 |
|  | R:GAAGGCGCCAGTGTTAGGAT |  |  |  |
| NPR-B | F:gtctggcttgagaagaagtagg | 102 | 60 | NM_001285674.1 |
|  | R:gtggagatgaagggaaaagg |  |  |  |
| NPR-C | F:CAAAGCATCAGGTGGCCTAGA | 202 | 60 | XM_018065682.1 |
|  | R:CACCGAAAAGTGGGATCGGA |  |  |  |
| β-casein | F:GTCCTCATCCTTGCCTGTCTG | 175 | 60 | XM_013964699.2 |
|  | R:GGAGTTCATCCTCTGTTTGCTG |  |  |  |
| IL-6 | F:CTTCAGTCCACTCGCTGTCT | 114 | 60 | HM565937.1 |
|  | R:AGTAGTCTGCTTGGGGTGGT |  |  |  |
| IL-1β | F:GGCTCCATGGGAGATGGAAA | 127 | 60 | XM_013967700.2 |
|  | R:AGACATGTTCGTAGGCACGG |  |  |  |
| TNF-α | F:GCATGAGCACCAAAAGCATGA | 198 | 60 | NM_001286442.1 |
|  | R:CTGGGGACTGCTCTTCCCTCT |  |  |  |
| ACACA | F:TCAGAGCTTCTGAGCTTGCC |  |  | XM_018064174.1 |
|  | R:AGGATCTACCCAGGCCACAT | 125 | 60 |  |
| SREBF1 | F:CGCACGCTTGAGAAGATTGG | 147 | 60 | NM_001285755.1 |
|  | R:TCCGACGCTGATACGGAGA |  |  |  |
